# Time-optimal trajectory planning based on event-trigger and conditional proportional control

**DOI:** 10.1371/journal.pone.0273640

**Published:** 2023-01-30

**Authors:** Guangrong Chen, Ningze Wei, Lei Yan, HuaFeng Lu, Jin Li

**Affiliations:** 1 Robotics Research Center, Beijing Jiaotong University, Beijing, P.R. China; 2 Machinery Department of Patent Office, China National Intellectual Property Administration, Beijing, P.R. China; Beijing Institute of Technology, CHINA

## Abstract

Trajectory planning is an important issue for manipulators and robots. To get a optimal trajectory, many constraints including actuators specifications, motion range of joints, workspace limitations, etc, and many objectives including the shortest time, the shortest distance, the lowest energy consumption, the minimum oscillations, obstacles-avoiding, etc, should be considered both. In this paper, firstly, the forward kinematics and inverse kinematics of a five axis manipulator are deduced. And, a simple method to choose one appropriate solution from multi solutions of inverse kinematics is proposed. Secondly, an easy-implemented optimization method of trajectory planning is proposed based on seventh order polynomial interpolation, event-trigger mechanism and conditional proportional control (P control). The proposed optimization method can capture the time optimal trajectory, and the actuators specifications including velocity, acceleration of motor can be guaranteed as well. Thirdly, comparative simulations and experiments validate the effectiveness and efficiency of proposed optimization method. The research provides an insight for the application of trajectory optimization on the micro controller with low computing capability and high real-time performance requirement.

## 1 Introduction

Trajectory planning is moving from point A to point B while avoiding collisions over time. This can be computed in both discrete and continuous methods. Trajectory planning is a major area in robotics as it gives way to autonomous vehicles [1], gives motion trajectory to manipulators [2] or robots [3]. The goal of trajectory planning is to generate the reference inputs to the motion control system which ensures that the planned trajectories can be executed [4].

**Fig 1:**
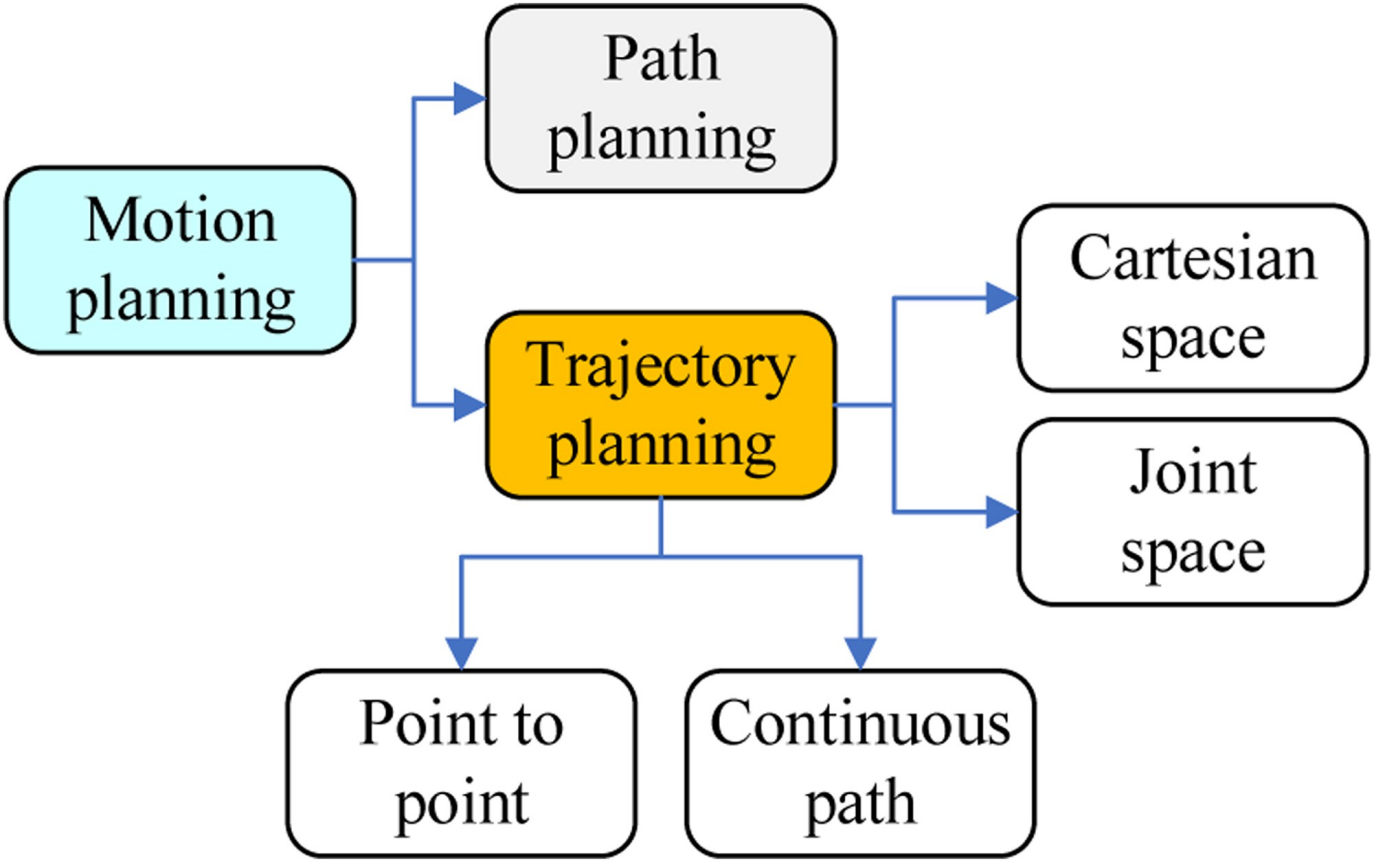
The relationships of trajectory planning.

Generally, motion planning [5] can be divided into path planning [6] and trajectory planning [7], as shown in Fig 1. The type of trajectory planning contains point-to-point path [8] and continuous path [9, 10], and the trajectory planning can be implemented in Cartesian space and joint space. More specifically, trajectory planning is sometimes referred to as motion planning and erroneously as path planning. Trajectory planning is distinct from path planning in that it is parametrized by time. Essentially trajectory planning encompasses path planning in addition to planning how to move based on velocity, time, and kinematics, etc.

There are many methods can be utilized to do the trajectory planning. Volkov YS studied the general problem of interpolation by polynomial splines and considered the construction of such splines using the coefficients of expansion of a certain derivative in B-splines. Then, the properties of the obtained systems of equations are analyzed and the interpolation error is estimated [11]. Farid G et al discussed the waypoints-based trajectory generation algorithm specifically for a quadrotor UAV and considers linear interpolation along with parabolic blends to achieve high computational efficiency and continue waypoints [12]. Based on the cubic trigonometric B-spline interpolation algorithm (CTB), Li H et al proposed a new improved method: CTB-EMD, which combines the cubic trigonometric B-spline interpolation algorithm (CTB) and empirical mode decomposition (EMD). In this method, the interpolation curve is more flexible because of the adjustability of shape of the cubic trigonometric B-splines curve. Thus, the overshoot and undershoot problems in the cubic spline interpolation curve can be avoided, and then the decomposition of the signal is more accurate and effect [13]. Fang Y et al proposed a smooth and time-optimal S-curve trajectory planning method to meet the requirements of high-speed and ultra-precision operation for robotic manipulators in modern industrial applications. This method utilizes a piecewise sigmoid function to establish a jerk profile with suitably chosen phase durations such that the generated trajectories are infinitely continuously differentiable under the given constraints on velocity, acceleration and jerk [14].

Among the above methods, the most commonly used is polynomial spline interpolation. However, the generated trajectory planning will be various if different order of polynomial in the spline interpolation is used. To solve this problem, many kinds of optimization methods of trajectory planning are investigated. Ju H et al proposed a time-optimal 3-5-3 polynomial interpolation trajectory planning algorithm based on Genetic Algorithm(GA) according to the velocity limitation of manipulator [15]. Liu Y et al presented a new method of online planning high smooth and time-optimal trajectory for robotic manipulators that applies an adaptive elite genetic algorithm with singularity avoidance (AEGA-SA) [16]. Fu R et al proposed a time-optimal 3-5-3 polynomial interpolation trajectory planning algorithm based on particle swarm optimization(PSO) according to the kinematic constraints of manipulator. The algorithm solves the problem that polynomial interpolation based trajectory planning is hard to be optimized by traditional optimization methods for its shortcomings of high order and lack of convex hull property, etc [17]. Yu L et al presented a general method for the trajectory planning of the redundant planar manipulator. The new application of knot points in the quintic B-spline curve is introduced to generate inverse solutions of class I redundant joints and the particle swarm optimization algorithm is extended to generate solutions of class II redundant joints [18]. Barnett E et al introduced a novel technique, called the bisection algorithm (BA), which is fully implemented in C++ and extends dynamic programming approaches to the problem. This approach is made feasible through careful control of the numerical integration process and the use of a bisection algorithm to resolve constraint violations during integration [19]. Guo H et al presented a simultaneous trajectory planning and tracking controller for use under cruise conditions based on a model predictive control (MPC) approach to address obstacle avoidance for an intelligent vehicle [20].

To get a optimal trajectory, many constraints including actuators specifications (kinematics and dynamics) [21, 22], motion range of joints, workspace limitations, etc and many objectives including the shortest time, the shortest distance, the lowest energy consumption, the minimum oscillations [23], obstacles-avoiding [24], etc, should be considered both. Ma J et al reported on a bounded control law for nonholonomic systems of unicycle-type that satisfactorily drive a vehicle along a desired trajectory while guaranteeing a minimum safe distance from another vehicle or obstacle at all times. The control law is comprised of two parts. The first is a trajectory tracking and set-point stabilization control law that accounts for the vehicle’s kinematic and dynamic constraints (i.e. restrictions on velocity and acceleration), and the second part is a real-time avoidance control law that guarantees collision-free transit for the vehicle in noncooperative and cooperative scenarios independently of bounded uncertainties and errors in the obstacles’ detection process [25]. Wang H et al proposed a smooth point-to-point trajectory planning method for industrial robots. In the accelerated part and decelerated part, the acceleration is planned with fourth-order polynomial formed with the property of the root multiplicity. Then near time-optimal trajectory can be obtained by maximizing the constant velocity part under kinematical constraints [26]. Rodríguez-Seda EJ et al described a new convex optimization (CO) approach to time-optimal trajectory planning (TOTP), which considers both torque and jerk limits. The key insight of the approach is that the non-convex jerk limits are transformed to linear acceleration constraints and indirectly introduced into CO as the linear acceleration constraints [22]. Hsu Y et al proposed a reinforcement learning approach of collision avoidance and investigate optimal trajectory planning for unmanned aerial vehicle (UAV) communication networks [27]. Wang W et al presented an improved artificial potential field method of trajectory planning and obstacle avoidance for redundant manipulators. The method not only focused on the position for the manipulator end-effectors but also considered their posture in the course of trajectory planning and obstacle avoidance [28]. Zhang Z et al presented a hierarchical three-layer trajectory planning framework to realize real-time collision avoidance under complex driving conditions. This is mainly ascribed to the generation of a collision-free trajectory cluster based on the speed and the path re-planning [29]. Wang C et al proposed an enabling motion planning method for post-impact situations by combining the polynomial curve and artificial potential field while considering obstacle avoidance. Then, a hierarchical controller that consists of an upper (a time-varying linear quadratic regulator) and a lower (a nonlinear-optimization-based torque allocation algorithm) controller is developed to track the planned motion [26].

Specially, time-optimal trajectory planning is a more common optimization type. Reiter A et al addressed a time-optimal path following along a predefined end-effector path for kinematically redundant robots, where nonredundant robots are included as special cases [30]. Lu L et al reported a time-optimal motion planning method for robotic machining of sculptured surfaces. As there are high requirements for tool path following accuracy, an efficient numerical integration method based on the Pontryagin maximum principle is adopted as the solver for the time-optimal tool motion planning problem in robotic machining [31]. Xidias EK et al proposed a novel approach based on Genetic Algorithm for time-optimal trajectory planning of a hyper-redundant manipulator which is requested to move from an initial configuration to a final configuration in 3D workspaces considering simultaneously the kinematical constraints of the manipulator (specifically velocity and acceleration) and the presence of obstacles [32].

And, real-time trajectory planning has been a research hotspot so far due to its superior dynamic adaptability [33]. Kim J et al presented a novel trajectory planning approach suitable for online industrial robot applications along short path segments such as spot-welding. The proposed method generates trajectories that are computationally efficient, dynamically near time-optimal, and maintain path-following integrity in high-frequency planning-and-control cycles [9]. Chai R et al developed a two-step strategy for real-time trajectory planning of a hypersonic vehicle (HV) in the reentry phase. The first step generates the optimal trajectory for the HV using a recently proposed fuzzy multiobjective transcription method. In the second step, the optimally generated trajectories are utilized to train a deep neural network (DNN), which is then acted as the optimal command generator in real time [33].

However, most of optimization methods of trajectory planning are based on iterative principle. That means it will cost many time in the optimization process and the optimization will cost lots of capacity of calculation of controller as well. The problem limits the application of these iterative optimization methods on some controller with low computing capability or some scenarios with real-time requirements [16]. Therefore, an easy-implemented optimization method of trajectory planning based on event-trigger and conditional P control is proposed to address the issue, where P control is short for proportional control, which comes from PID control (Proportional-Integral-Derivative control) [34] and conditional means that P control works when some conditions are satisfied. As for event-trigger, Liu J et al proposed an event-triggered vehicle-following control scheme for connected and automated vehicles (CAVs) considering nonideal Vehicle-to-Vehicle communications such as communication delays and packet dropouts. An output-based event-triggered mechanism is employed for reducing computational burden. An Event-Triggered Model Predictive Control (ETMPC) is proposed by combining with a multi-target controller for the lateral and longitudinal vehicle-following control of CAVs [35]. Ding X et al proposed an enabling event-triggered sideslip angle estimator by using the kinematic information from a low-cost global positioning system (GPS) and an on-board inertial measurement unit (IMU) [36].

The paper is organized as follows. In *Section* 2, the system model of a five axis manipulator are analyzed. In *Section* 3, an easy-implemented optimization method of trajectory planning is proposed based on seventh order polynomial interpolation, event-trigger mechanism and conditional P control. In *Section* 4, comparative simulations and experiments are implemented to validate the effectiveness and efficiency of proposed optimization method. In *Section* 5, conclusions are drawn and future works are issued. In *Appendix* *A* and *Appendix* *B*, the forward kinematics and inverse kinematics of a five axis manipulator are deduced, respectively. In *Appendix* *C* gives out why the planning time can be used to adjust the maximum values of planning velocity and acceleration. In *Appendix* *D* gives out why the P control could guarantee the stability of time optimal controller and the converge of optimization.

## 2 Model

**Fig 2:**
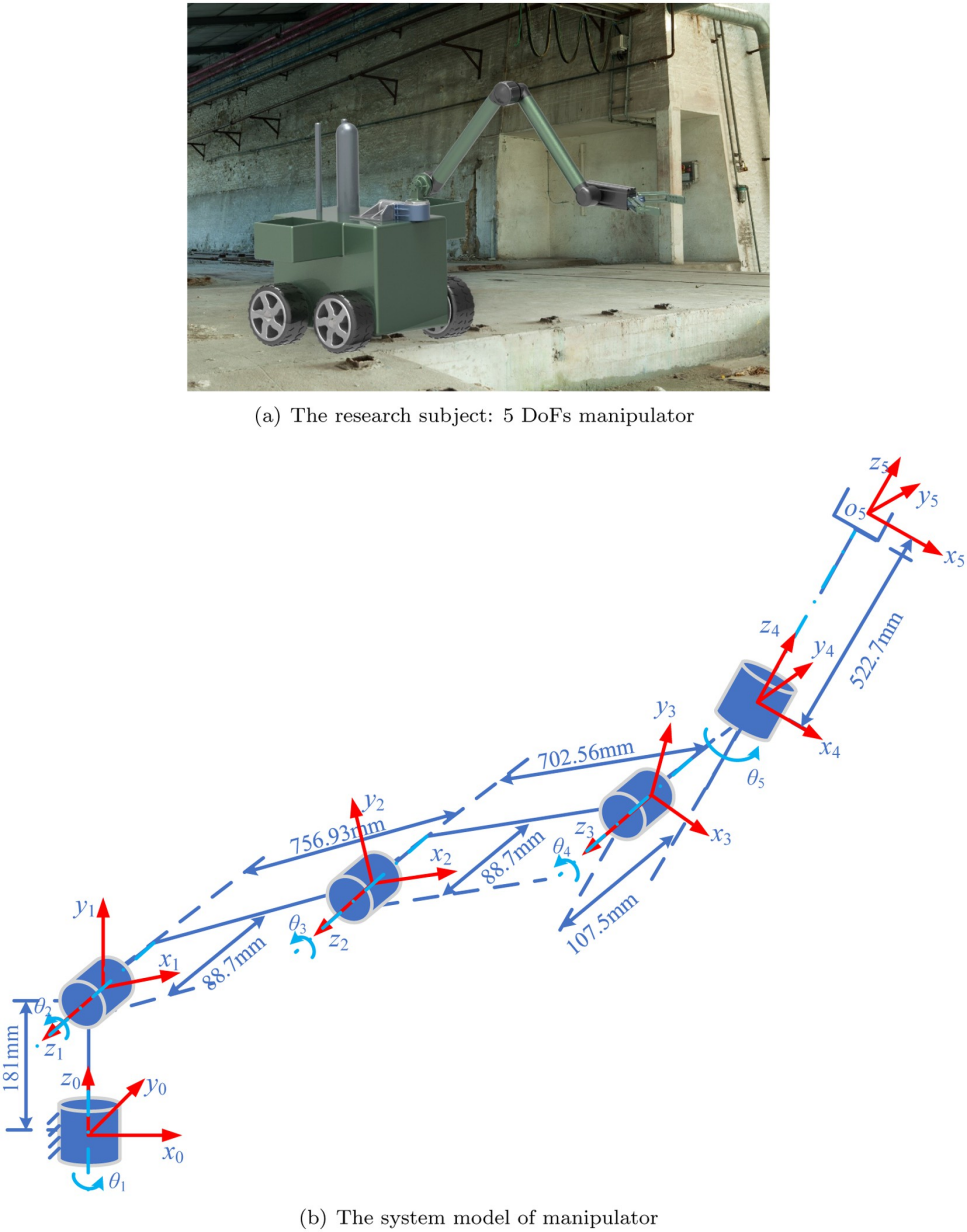
System model. (a) The research subject: 5 DoFs manipulator. (b) The system model of manipulator.

The research subject: a 6 degrees of freedom (DoFs) manipulator with 5 axis and its corresponding system model are shown in Fig 2 and 2(b), respectively, where coordinate systems {0}, {1}, {2}, {3}, {4} are established on the 5 actuators of 5 DoFs, and coordinate systems {5} is established on the grasper at the end effector. Actually, there exists extra 1 DoF in the grasper at the end effector. Since the DoF is for grasping task and it does not affect the trajectory planning of manipulator, only 5 DoFs of manipulator are considered in the following. The link relationship parameters of the manipulator mainly contain: the length of the link *a*_*i*_, the joint angle *θ*_*i*_, the offset of the link *d*_*i*_ and the torsion angle of the link *α*_*i*_, where *i* = 1, 2, 3, 4, 5. The four parameters are the DH parameters of the manipulator. According to the geometric model of the manipulator, its D-H parameters can be determined as shown in Table 1. The detailed forward kinematics and inverse kinematics are given out in the Appendices A and B, respectively. Specially, the forward kinematics is unique, while there are eight solutions of inverse kinematics.

**Table 1:** DH parameters of manipulator.

No.	*θ*_*i*_	*d*_*i*_	*a*_*i*_	*α*_*i*_
1	*θ*_1_	181 mm	0	90°
2	*θ*_2_	-88.7 mm	756.93 mm	0
3	*θ*_3_	-88.7 mm	702.56 mm	0
4	*θ*_4_	-107.5 mm	0	-90°
5	*θ*_5_	522.7 mm	0	0

## 3 Trajectory planning and optimization

### 3.1 Trajectory planning based on polynomial interpolation

Take the trajectory planning of point-to-point path in Cartesian space as an example, the trajectory planning based on fifth order polynomial interpolation can be employed to satisfy the six constrains, that is, the position, velocity and acceleration of end effector of manipulator at the start knot point and final knot point are all should be zero and differentiable (they can be non-zero in continuous path). The trajectory planning based on fifth order polynomial interpolation only can get continuous position and velocity from start knot point to final knot point. In order to make the acceleration continuous and differentiable as well to avoid the oscillations caused by sudden accelerations when the manipulator starts and stops, the trajectory planning based on seventh order polynomial interpolation can be utilized to address the issue if the jerk at the start knot point and final knot point are both constrained to be zero.

The trajectory planning based on seventh order polynomial interpolation can be written as

$$\begin{array}{c} \begin{array}{c} p(t)=c_{0}+c_{1}t+c_{2}t^{2}+c_{3}t^{3}+c_{4}t^{4}+c_{5}t^{5}+c_{6}t^{6}+c_{7}t^{7}\\ \dot{p}(t)=c_{1}+2c_{2}t+3c_{3}t^{2}+4c_{4}t^{3}+5c_{5}t^{4}+6c_{6}t^{5}+7c_{7}t^{6}\\ \ddot{p}(t)=2c_{2}+6c_{3}t+12c_{4}t^{2}+20c_{5}t^{3}+30c_{6}t^{4}+42c_{7}t^{5}\\ \dddot{p}(t)=6c_{3}+24c_{4}t+60c_{5}t^{2}+120c_{6}t^{3}+210c_{7}t^{4} \end{array} \end{array}$$ (1)

where $p,\dot{p},\ddot{p},\dddot{p}$ are the position, velocity, acceleration and jerk of end effector of manipulator, respectively. *c*_*i*_, *i* = 0, …, 7 are the coefficients of the seventh order polynomial.

Then the constraint conditions, i.e. the position, velocity, acceleration and jerk of end effector of manipulator at the start knot point *p*_0_ and final knot point *p*_0_ are respectively

$$\begin{array}{c} \left\{ \begin{array}{c} p(t_{0})=p_{0}\\ \dot{p}(t_{0})={\dot{p}}_{0}\\ \ddot{p}(t_{0})={\ddot{p}}_{0}\\ \dddot{p}(t_{0})={\dddot{p}}_{0}\\ p(t_{\text{f}})=p_{\text{f}}\\ \dot{p}(t_{\text{f}})={\dot{p}}_{\text{f}}\\ \ddot{p}(t_{\text{f}})={\ddot{p}}_{\text{f}}\\ \dddot{p}(t_{\text{f}})={\dddot{p}}_{\text{f}} \end{array}\right. \end{array}$$ (2)

where *t*_0_, *t*_f_ are the known time passing through the start knot point and final knot point, respectively. The subscript {0, f} represent the known variables at the start knot point and final knot point, respectively. Specially, ${\dot{p}}_{0}={\ddot{p}}_{0}={\dddot{p}}_{0}={\dot{p}}_{\text{f}}={\ddot{p}}_{\text{f}}={\dddot{p}}_{\text{f}}=0$ in the trajectory planning of point-to-point path.

Substituting (2) into (1), the coefficients of the seventh order polynomial can be obtained as

$$\begin{array}{c} \left\{ \begin{array}{c} c_{0}=p_{0}\\ c_{1}={\dot{p}}_{0}\\ c_{2}=\frac{1}{2}{\ddot{p}}_{0}\\ c_{3}=\frac{1}{6}{\dddot{p}}_{0}\\ c_{4}=\frac{210(p_{\text{f}}-p_{0})-t_{\text{f}}[(30{\ddot{p}}_{0}-15{\ddot{p}}_{\text{f}})+(4{\dddot{p}}_{0}+{\dddot{p}}_{\text{f}})t_{\text{f}}^{2}+120{\dot{p}}_{0}+90{\dot{p}}_{\text{f}}]}{6t_{\text{f}}^{4}}\\ c_{5}=\frac{-168(p_{\text{f}}-p_{0})-t_{\text{f}}[(20{\ddot{p}}_{0}-14{\ddot{p}}_{\text{f}})t_{\text{f}}+(2{\dddot{p}}_{0}+{\dddot{p}}_{\text{f}})t_{\text{f}}^{2}+90{\dot{p}}_{0}+78{\dot{p}}_{\text{f}}]}{2t_{\text{f}}^{5}}\\ c_{6}=\frac{420(p_{\text{f}}-p_{0})-t_{\text{f}}[(45{\ddot{p}}_{0}-39{\ddot{p}}_{\text{f}})t_{\text{f}}+(4{\dddot{p}}_{0}+3{\dddot{p}}_{\text{f}})t_{\text{f}}^{2}+216{\dot{p}}_{0}+204{\dot{p}}_{\text{f}}]}{6t_{\text{f}}^{6}}\\ c_{7}=\frac{-120(p_{\text{f}}-p_{0})-t_{\text{f}}[(12{\ddot{p}}_{0}-12{\ddot{p}}_{\text{f}})t_{\text{f}}+({\dddot{p}}_{0}+{\dddot{p}}_{\text{f}})t_{\text{f}}^{2}+60{\dot{p}}_{0}+60{\dot{p}}_{\text{f}}]}{6t_{\text{f}}^{7}} \end{array}\right. \end{array}$$ (3)

As thus, the trajectory planning of point-to-point path in Cartesian space with constrains, specifying that the position, velocity, acceleration and jerk of end effector of manipulator at the start knot point and final knot point are continuous from zero, is handled. However, the obtained trajectory is not optimal since the planning time is pre-set and it can be optimized.

### 3.2 Trajectory optimization

After trajectory planning, there are two steps to achieve the trajectory Optimization. The first step is to obtain one appropriate solution of inverse kinematics from the eight calculating solutions Eqs (19), (20) and (23)–(25) based on the current configuration/pose of manipulator. The second step is to seek for the optimal planning time satisfying the actuators specifications including velocity, acceleration of motor.

#### 3.2.1 Appropriate solution of inverse kinematics

In the first step, the real-time joint angles $\theta =[\begin{array}{ccccc} \theta_{1} & \theta_{2} & \theta_{3} & \theta_{4} & \theta_{5} \end{array}]$ can be measured by sensors firstly. Then the position and attitude parameters $p=[\begin{array}{cccccc} x & y & z & \alpha & \beta & \gamma \end{array}]$ of end effector of manipulator can be obtained by forward kinematics Eqs (8)–(11). By using *i*-th *i* = 1, (*i* ∈ [1, 8]) solution of inverse kinematics, the corresponding joint angles are $\theta^{i}=[\begin{array}{ccccc} \theta_{1}^{i} & \theta_{2}^{i} & \theta_{3}^{i} & \theta_{4}^{i} & \theta_{5}^{i} \end{array}]$. If the difference between the actual angles *θ* and the calculating angles *θ*_*i*_ through forward and inverse kinematics satisfies

$$\begin{array}{c} |\theta^{i}-\theta |\leq \varepsilon_{\theta } \end{array}$$ (4)

where *ε*_*θ*_ is the self-determined angle margin to distinguish the appropriate solution of inverse kinematics from the eight calculating solutions Eqs (19), (20) and (23)–(25), then the *i*-th solution of inverse kinematics is the appropriate solution.

#### 3.2.2 Event-trigger mechanism

The trajectory planning Eq (1) should guarantee the planning velocity and acceleration are respectively within the maximum velocity and acceleration of actuators specifications. Compare the maximum values of planning velocity and acceleration with the maximum velocity and acceleration of actuators specifications, respectively, 5 events can be obtained as follows.

**Event 1**: The maximum value of planning velocity is smaller than the maximum velocity of actuators specifications and the maximum value of planning acceleration is smaller than the maximum acceleration of actuators specifications. In this event, the trajectory planning Eq (1) should be adjusted to increase the maximum value of planning velocity until it reaches to its corresponding maximum velocity of actuators specifications, or to increase the maximum value of planning acceleration until it reaches to its corresponding maximum acceleration of actuators specifications. Noting that the maximum values of planning velocity and acceleration cannot reach to their corresponding maximum velocity and acceleration of actuators specifications simultaneously.

**Event 2**: The maximum value of planning velocity is larger than the maximum velocity of actuators specifications, while the maximum value of planning acceleration is smaller than the maximum acceleration of actuators specifications. In this event, the trajectory planning Eq (1) should be adjusted to decrease the maximum value of planning velocity until it reaches to its maximum value of actuators specifications. The maximum value of planning acceleration can not be taken into consideration since it is in its maximum values of actuators specifications.

**Event 3**: The maximum value of planning velocity is smaller than the maximum velocity of actuators specifications, while the maximum value of planning acceleration is larger than the maximum acceleration of actuators specifications. In this event, the trajectory planning Eq (1) should be adjusted to decrease the maximum value of planning acceleration until it reaches to its maximum value of actuators specifications. The maximum value of planning velocity can not be taken into consideration since it is in its maximum values of actuators specifications.

**Event 4**: The maximum values of planning velocity and acceleration are both larger than the maximum velocity and acceleration of actuators specifications. In this event, the trajectory planning Eq (1) should be adjusted to decrease the maximum values of planning velocity and acceleration until one of them reaches to its maximum value of actuators specifications and the other is smaller than its maximum value of actuators specifications, or both of them reaches to their related maximum values of actuators specifications at the same time.

**Event 5**: The maximum values of planning velocity and acceleration are both at the maximum velocity and acceleration of actuators specifications, or one of them at its maximum value of actuators specifications. In this event, nothing should be done since the adjustment of trajectory planning Eq (1) will affect the maximum values of planning velocity and acceleration simultaneously.

It is easy to find that the adjustment of trajectory planning Eq (1) is event-based, and the event-trigger mechanism is clear.

#### 3.2.3 Time optimal controller based on conditional P control

Before designing the time optimal controller, two problems should be clear: 1) which element can be used to adjust the maximum values of planning velocity and acceleration efficiently (what is the control input); and 2) how to use the element to do the adjustment (how to set the control law). To handle these two issues, two theorems are given out as follows.

**Theorem 1**: In the point to point trajectory planing based on polynomial interpolation, the planning time is in inverse proportion to the maximum velocity and acceleration of planning trajectory.

**Theorem 2**: Taking the zero errors between the maximum values of planning velocity and acceleration and the maximum velocity and acceleration of actuators specifications as the control and optimization objective, the P control could guarantee the stability of time optimal controller and the converge of optimization.

The proofs of Theorem 1 and Theorem 1 are given out in the Appendices C and D, respectively.

Based on the event-trigger mechanism, the detailed time optimal controller for every event can be rewritten as

$$\begin{array}{c} \begin{array}{c} T=t_{\text{f}}-t_{0}=\{\begin{array}{c} TOC1\\ TOC2\\ TOC3\\ TOC4\\ OPT \end{array}\\ =\{\begin{array}{cc} \begin{array}{c} T+k_{\dot{\theta }}(\text{max}\;\{|\dot{\theta }|\}-{\dot{\theta }}_{\text{max}})\\ +k_{\ddot{\theta }}(\text{max}\;\{|\ddot{\theta }|\}-{\ddot{\theta }}_{\text{max}}), \end{array} & Event1:\{\begin{array}{c} \text{max}\;\{|\dot{\theta }|\}<{\dot{\theta }}_{\text{max}}\\ \text{max}\;\{|\ddot{\theta }|\}<{\ddot{\theta }}_{\text{max}} \end{array}\\ T+k_{\dot{\theta }}(\text{max}\;\{|\dot{\theta }|\}-{\dot{\theta }}_{\text{max}}), & Event2:\{\begin{array}{c} \text{max}\;\{|\dot{\theta }|\}>{\dot{\theta }}_{\text{max}}\\ \text{max}\;\{|\ddot{\theta }|\}<{\ddot{\theta }}_{\text{max}} \end{array}\\ T+k_{\ddot{\theta }}(\text{max}\;\{|\ddot{\theta }|\}-{\ddot{\theta }}_{\text{max}}), & Event3:\{\begin{array}{c} \text{max}\;\{|\dot{\theta }|\}<{\dot{\theta }}_{\text{max}}\\ \text{max}\;\{|\ddot{\theta }|\}>{\ddot{\theta }}_{\text{max}} \end{array}\\ \begin{array}{c} T+k_{\dot{\theta }}(\text{max}\;\{|\dot{\theta }|\}-{\dot{\theta }}_{\text{max}})\\ +k_{\ddot{\theta }}(\text{max}\;\{|\ddot{\theta }|\}-{\ddot{\theta }}_{\text{max}}), \end{array} & Event4:\{\begin{array}{c} \text{max}\;\{|\dot{\theta }|\}>{\dot{\theta }}_{\text{max}}\\ \text{max}\;\{|\ddot{\theta }|\}>{\ddot{\theta }}_{\text{max}} \end{array}\\ T_{opt} & \begin{array}{c} Event5:\\ \left(\text{max}\;\{|\dot{\theta }|\}={\dot{\theta }}_{\text{max}}|\text{max}\{|\ddot{\theta }|\}={\ddot{\theta }}_{\text{max}}\right)\\ \&\{\begin{array}{c} \text{max}\;\{|\dot{\theta }|\}\leq {\dot{\theta }}_{\text{max}}\\ \text{max}\;\{|\ddot{\theta }|\}\leq {\ddot{\theta }}_{\text{max}} \end{array} \end{array} \end{array} \end{array} \end{array}$$ (5)

where *TOC*1, *TOC*2, *TOC*3, *TOC*4, *OPT* are the corresponding control laws of *Event*1, *Event*2, *Event*3, *Event*4, *Event*5, respectively. *T*_*opt*_ is the optimal planning time. $k_{\dot{\theta }},k_{\ddot{\theta }}$ are the tuning parameters of errors of velocity and acceleration in the time optimal controller, respectively. ${\dot{\theta }}_{\text{max}},{\ddot{\theta }}_{\text{max}}$ are the maximum velocity and acceleration of actuators specifications, respectively. Specially, ${\dot{\theta }}_{\text{max}},{\ddot{\theta }}_{\text{max}}$ can be individual maximum velocity and acceleration of actuators specifications for individual axis/joint.

Actually, there exist two cases in the second step. They can be concluded as follows.

**Case 1**: The planning velocity $\dot{\theta }$ and acceleration $\ddot{\theta }$ are both in the actuators specifications. In this case, however, the planning time may be not optimal, it could be too long. As thus, the planning time *T* = *t*_f_ − *t*_0_ can be decreased to close to the optimal planning time and the actuators specifications are satisfied simultaneously. This case only contains Event 1.

**Case 2**: The planning velocity $\dot{\theta }$ or acceleration $\ddot{\theta }$ is larger than that the actuators can provide. In this case, the simplest way to solve the problem is increasing the planning time *T* = *t*_f_ − *t*_0_. This case contains Event 2, 3, 4.

Event 5 is the end of time optimal controller and the optimal planning time *T*_*opt*_ will be obtained. To avoid the overshoot of the adjustment of trajectory planning Eq (1) based on P control, Case 1 should be set/judged before Case 2.

Based on the five events and two cases, in order to achieve the whole time optimal controller without using iterative algorithms, an easy-implemented optimization method for planning time *T* = *t*_f_ − *t*_0_ is proposed as

$$\begin{array}{c} \begin{array}{c} T=t_{\text{f}}-t_{0}\\ =\{\begin{array}{cc} \begin{array}{c} T+k_{\dot{\theta }}(\text{max}\;\{|\dot{\theta }|\}-{\dot{\theta }}_{\text{max}})(\text{max}\;\{|\dot{\theta }|\}<{\dot{\theta }}_{\text{max}})\\ +k_{\ddot{\theta }}(\text{max}\;\{|\ddot{\theta }|\}-{\ddot{\theta }}_{\text{max}})(\text{max}\;\{|\ddot{\theta }|\}<{\ddot{\theta }}_{\text{max}}), \end{array} & Case1\\ \begin{array}{c} T+k_{\dot{\theta }}(\text{max}\;\{|\dot{\theta }|\}-{\dot{\theta }}_{\text{max}})(\text{max}\;\{|\dot{\theta }|\}\geq {\dot{\theta }}_{\text{max}})\\ +k_{\ddot{\theta }}(\text{max}\;\{|\ddot{\theta }|\}-{\ddot{\theta }}_{\text{max}})(\text{max}\;\{|\ddot{\theta }|\}\geq {\ddot{\theta }}_{\text{max}}), \end{array} & Case2 \end{array} \end{array} \end{array}$$ (6)

where if the inequality in brackets holds, it is 1, otherwise it is 0.

**Remark**: Under ideal conditions, the planning velocity and acceleration equal to the maximum velocity and acceleration of actuators specifications, respectively, that is, $\dot{\theta }={\dot{\theta }}_{\text{max}}$ and $\ddot{\theta }={\ddot{\theta }}_{\text{max}}$ with the optimal planning time *T* = *t*_f_ − *t*_0_. It can be found that the time optimal controller Eq (6) is similar to P control in PID controller [34] and the P control is conditional. Whether P control is activated depends on whether the corresponding actuators specification is satisfied. That is why it is called conditional P control. Here, $k_{\dot{\theta }},k_{\ddot{\theta }}$ are similar to the proportional coefficient in PID control. Besides, other constrains contain: maximum jerk [37], kinematic and dynamic requirements, also can be considered into the right side of Eq (6). And, the optimal planning time in Eq (6) can be discretized to make it available to be used directly in practical applications. The resolution of the optimal planning time *T* can be the same as the servo control cycle Δ*T* = 0.001s.

When the optimal planning time *T* = *t*_f_ − *t*_0_ is determined, the optimized trajectory for end effector of manipulator $p,\dot{p},\ddot{p},\dddot{p}$ and joint angles $\theta ,\dot{\theta },\ddot{\theta },\dddot{\theta }$ can be output both for control.

### 3.3 Control scheme

**Fig 3:**
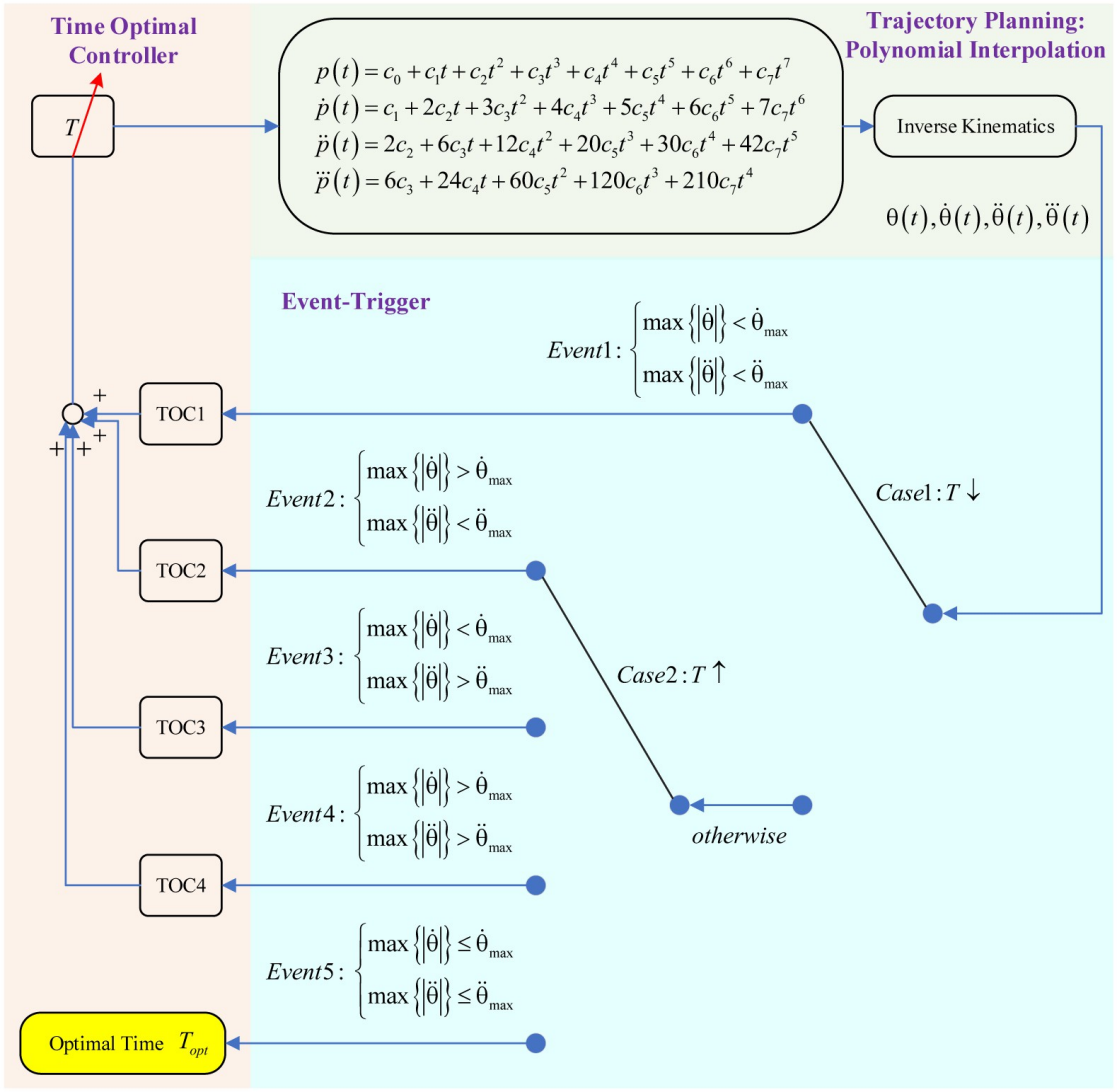
The whole control scheme of trajectory optimization.

The whole control scheme of trajectory optimization is shown in Fig 3. The control scheme contains three parts. The first part is a trajectory planning based on polynomial interpolation and it is similar to the research subject. The second part is an event-trigger module and it is similar to the feedback loop. The third part is a time optimal controller based on P control. The three parts form a closed loop to search for the optimal planning time and there is no input essentially.

### 3.4 Flow chart

**Fig 4:**
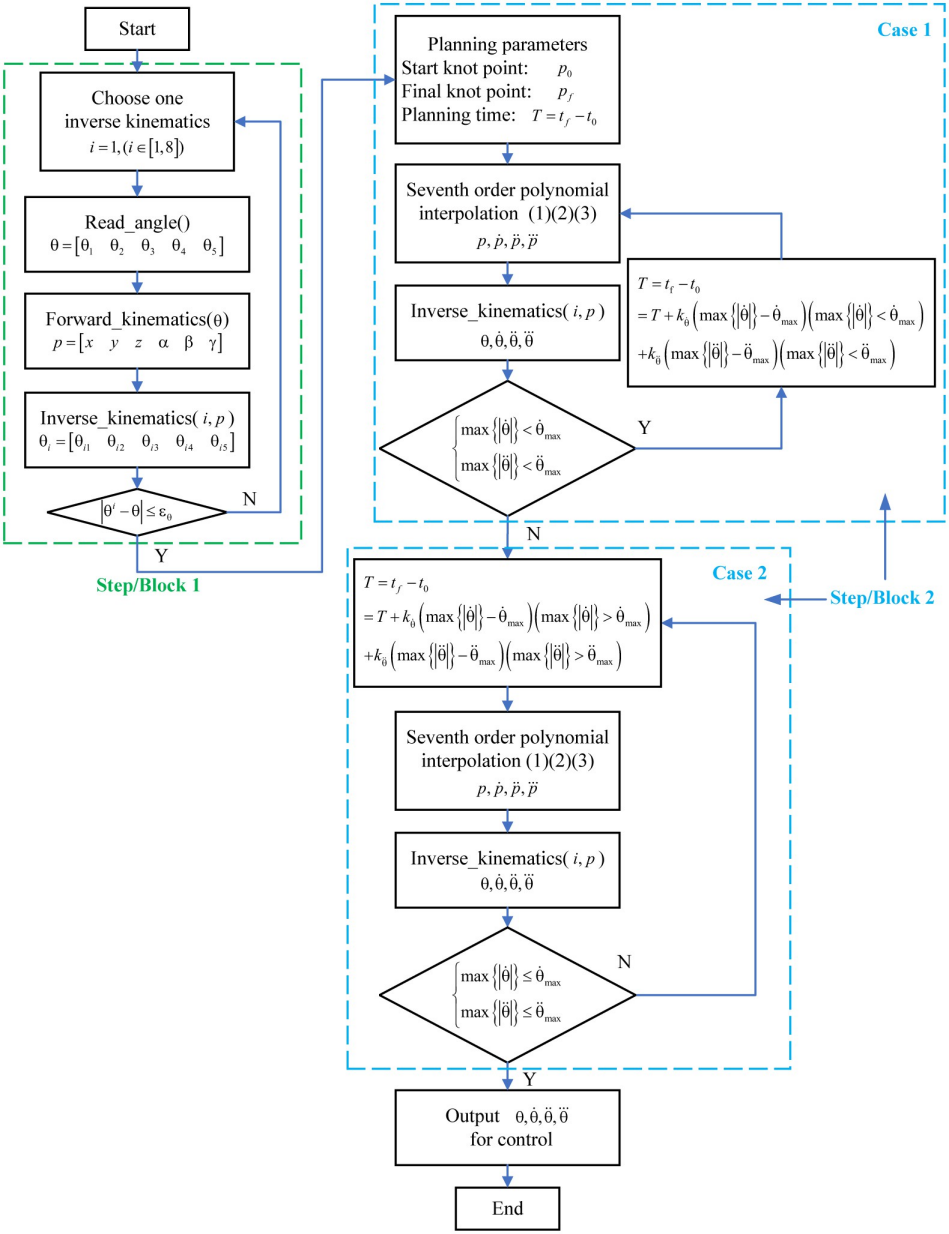
The program flow chart of trajectory optimization.

Based on the proposed optimization method of trajectory planning, the program flow chart is shown in Fig 4. The program flow chart contains two blocks that are consistent with the two steps in trajectory optimization. The first block is to choose the appropriate solution for inverse kinematics, and the second block considering two cases is to search for the optimal planning time within actuators specifications. The P-like control algorithm has more concise and efficient structure without considering complicated iterative process. Therefore, it can be easily implemented on micro controller unit with low computing capability.

## 4 Validations

### 4.1 Simulations

To validate the effectiveness and efficiency of proposed optimization method, comparative simulations are implemented. The simulation parameters are shown in Table 2. The simulation sets of point to point trajectory planning [21] are shown in Table 3. Specially, the actuators specifications including the limitations of velocity and acceleration of motor and they are ${\ddot{\theta }}_{\text{max}}=0.08{\text{rad}/\text{s}}^{2}$ and ${\dot{\theta }}_{\text{max}}=0.12\text{rad}/\text{s}$, respectively. And the limitations are for inputs of joints so that the actuator may contain a reducer.

**Table 2:** Simulation parameters.

Parameter	Value
$p_{0}=[\begin{array}{c} x_{0}\\ y_{0}\\ z_{0}\\ \alpha_{0}\\ \beta_{0}\\ \gamma_{0} \end{array}]$	$\left[\begin{array}{c} 1465.87\text{mm}\\ 547.77\text{mm}\\ -202.12\text{mm}\\ 0.0305\text{rad}\\ -2.9697\text{rad}\\ -2.7899\text{rad} \end{array}\right]$
$p_{\text{f}}=[\begin{array}{c} x_{\text{f}}\\ y_{\text{f}}\\ z_{\text{f}}\\ \alpha_{\text{f}}\\ \beta_{\text{f}}\\ \gamma_{\text{f}} \end{array}]$	$\left[\begin{array}{c} 510.41\text{mm}\\ 1453.86\text{mm}\\ 482.58\text{mm}\\ 0.2794\text{rad}\\ -2.4930\text{rad}\\ -1.6509\text{rad} \end{array}\right]$
*ε*_*θ*_	0.001rad
Simulation step size	0.001s
Simulation software	Matlab(R2018b)
Solver	ode45(Dormand-Prince)
Computer system	Windows 10, 64-bit
CPU	Intel(R) Core(TM) i7-8750H 2.2GHz
RAM	16GB

**Table 3:** Simulation sets.

Simulations	Order of polynomial interpolation	$\left[\begin{array}{c} T=t_{\text{f}}\\ t_{0} \end{array}\right]$	$\left[\begin{array}{c} {\dot{\theta }}_{\text{max}}\\ {\ddot{\theta }}_{\text{max}}\\ k_{\dot{\theta }}\\ k_{\ddot{\theta }} \end{array}\right]$	$\begin{array}{c} \text{Start}\;\text{knot}\;\text{point}\\ \to \\ \text{Final}\;\text{knot}\;\text{point} \end{array}$
Simulation 1	5th	$\left[\begin{array}{c} 10\text{s}\\ 0 \end{array}\right]$	Null	*p*_0_ → *p*_f_
Simulation 2	5th	$\left[\begin{array}{c} 20\text{s}\\ 0 \end{array}\right]$	Null	*p*_0_ → *p*_f_
Simulation 3	7th	$\left[\begin{array}{c} 10\text{s}\\ 0 \end{array}\right]$	Null	*p*_f_ → *p*_0_
Simulation 4	7th	$\left[\begin{array}{c} 20\text{s}\\ 0 \end{array}\right]$	Null	*p*_f_ → *p*_0_
Simulation 5	7th	$\left[\begin{array}{c} \text{TBD}\\ 0 \end{array}\right]$	$\left[\begin{array}{c} 0.12\text{rad}/\text{s}\\ 0.08{\text{rad}/\text{s}}^{2}\\ 83\\ 83 \end{array}\right]$	*p*_0_ → *p*_f_
Simulation 6	7th	$\left[\begin{array}{c} \text{TBD}\\ 0 \end{array}\right]$	$\left[\begin{array}{c} 0.12\text{rad}/\text{s}\\ 0.02{\text{rad}/\text{s}}^{2}\\ 9\\ 162.1 \end{array}\right]$	*p*_f_ → *p*_0_

**Fig 5:**
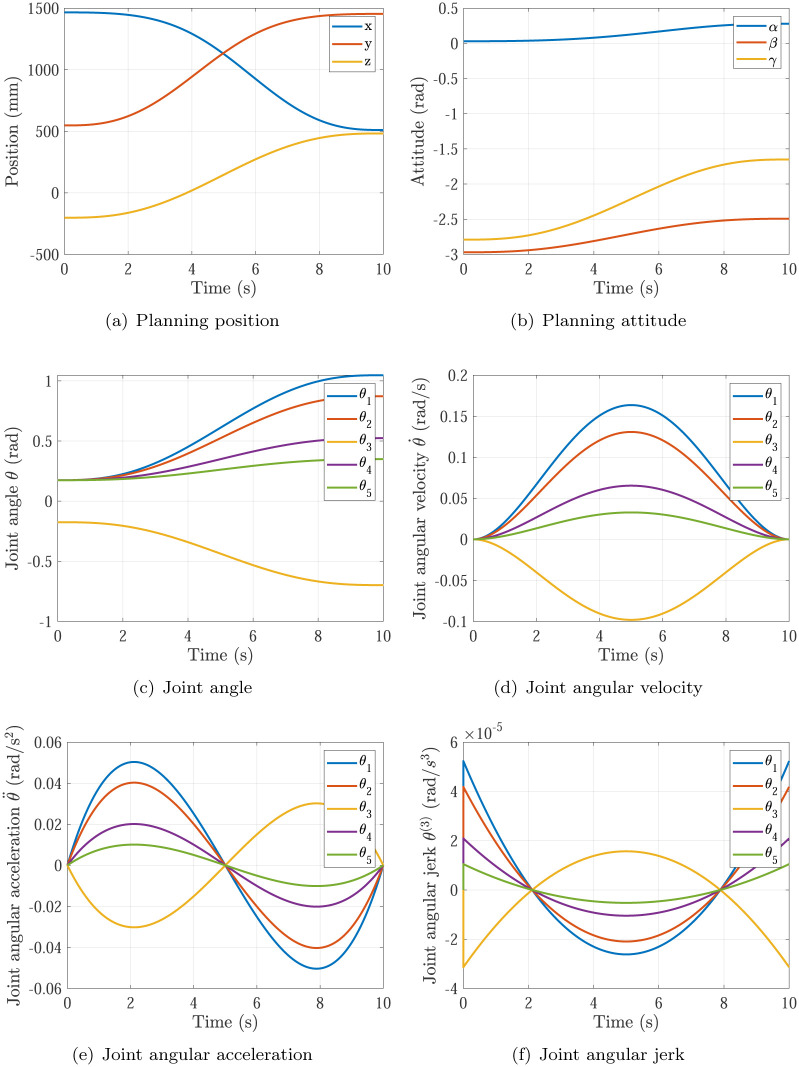
The trajectory planning results of Simulation 1 based on fifth order polynomial interpolation. (a) Planning position, (b) Planning attitude, (c) Joint angle, (d) Joint angular velocity, (e) Joint angular acceleration, (f) Joint angular jerk.

The trajectory planning results of Simulation 1∼6 are shown in Figs 5∼10, respectively. The comparisons of simulations in Figs 5∼10 are shown in Table 4.

**Fig 6:**
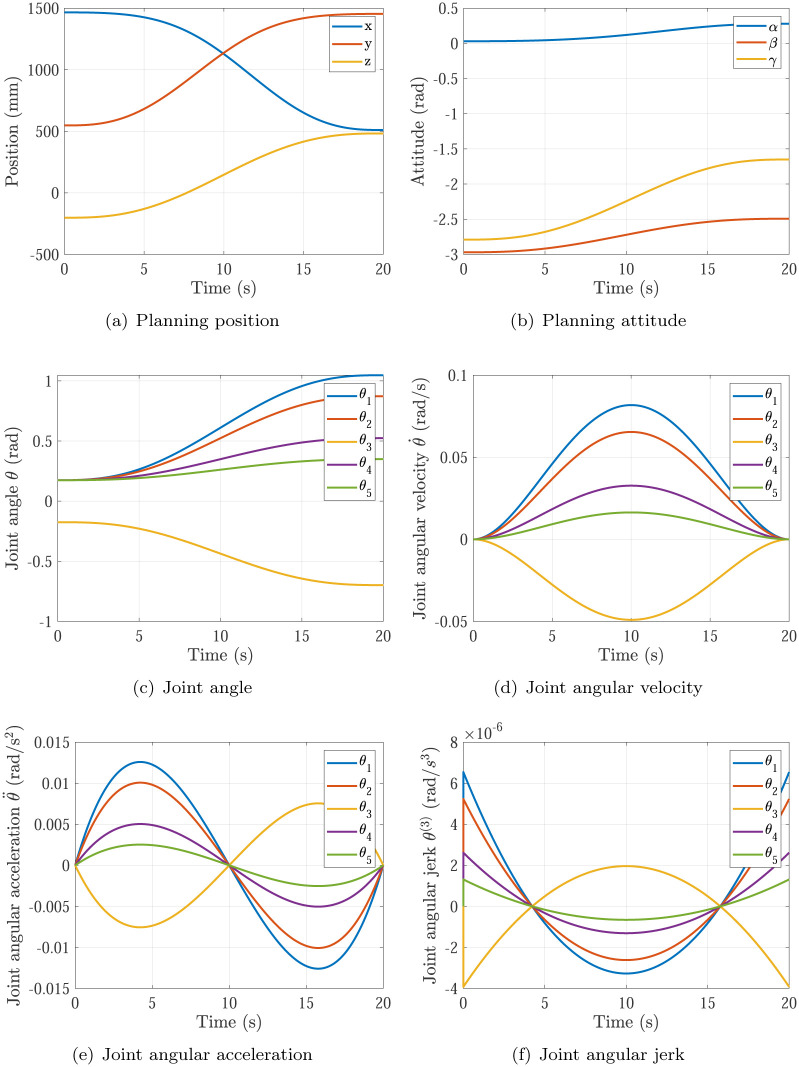
The trajectory planning results of Simulation 2 based on fifth order polynomial interpolation. (a) Planning position, (b) Planning attitude, (c) Joint angle, (d) Joint angular velocity, (e) Joint angular acceleration, (f) Joint angular jerk.

**Fig 7:**
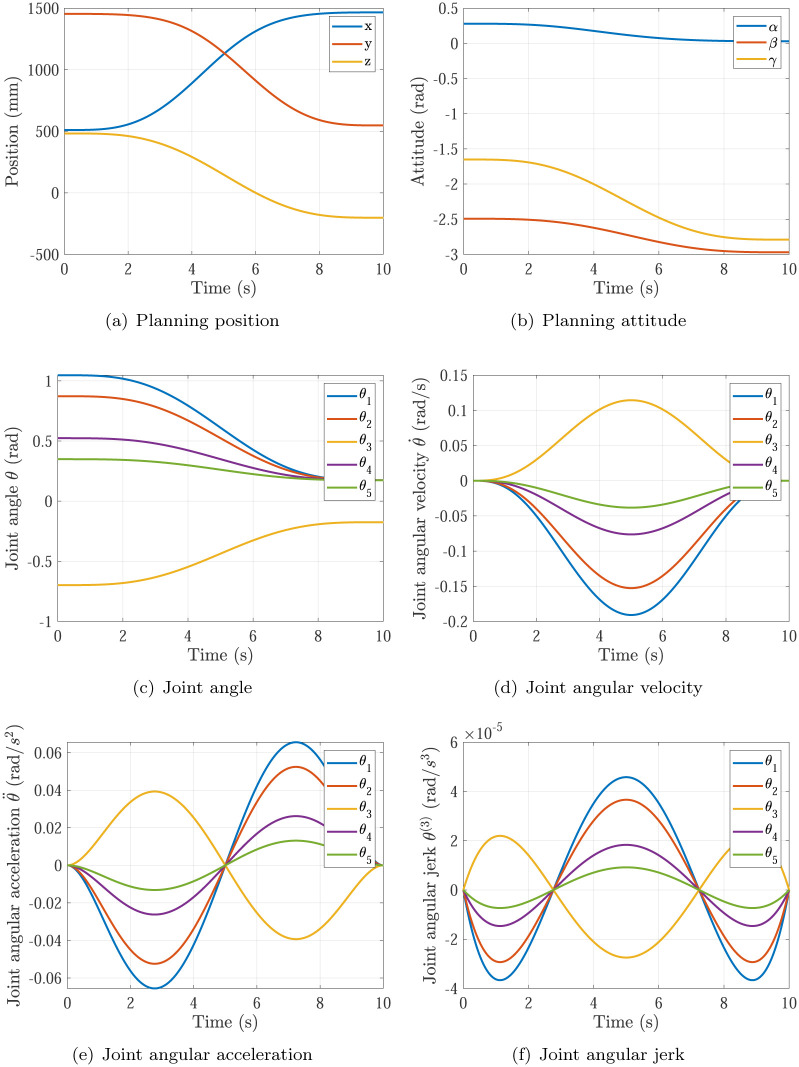
The trajectory planning results of Simulation 3 based on fifth order polynomial interpolation. (a) Planning position, (b) Planning attitude, (c) Joint angle, (d) Joint angular velocity, (e) Joint angular acceleration, (f) Joint angular jerk.

**Fig 8:**
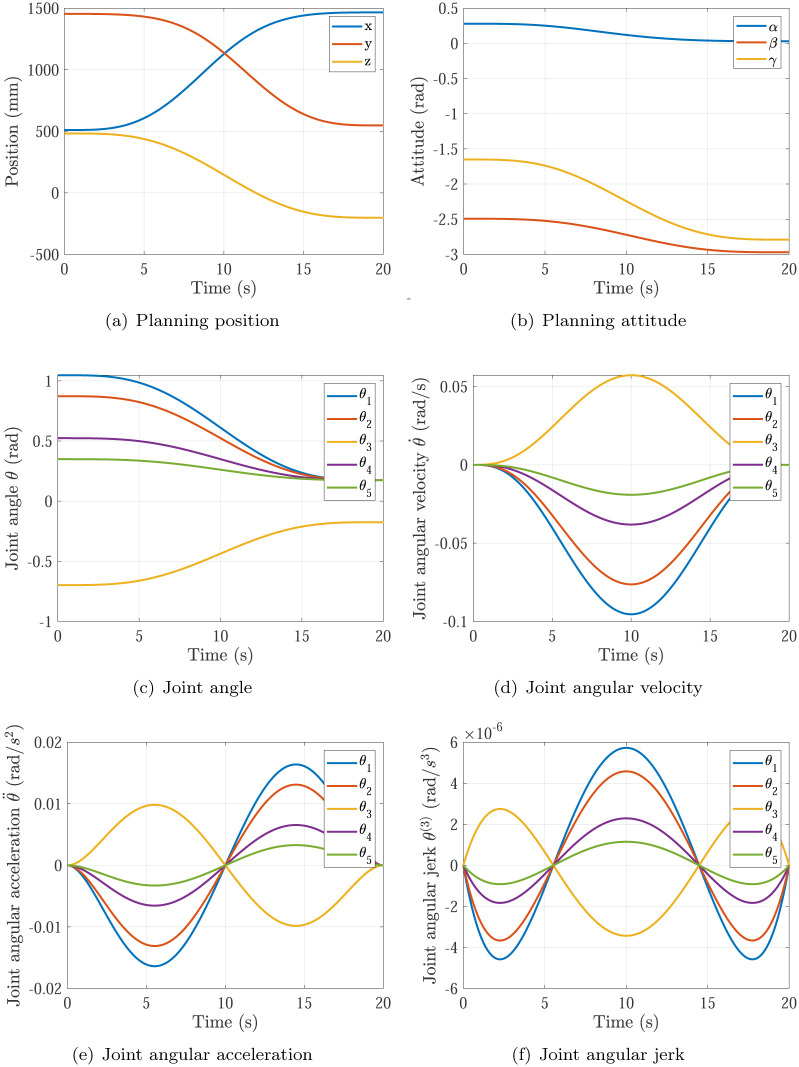
The trajectory planning results of Simulation 4 based on fifth order polynomial interpolation. (a) Planning position, (b) Planning attitude, (c) Joint angle, (d) Joint angular velocity, (e) Joint angular acceleration, (f) Joint angular jerk.

**Fig 9:**
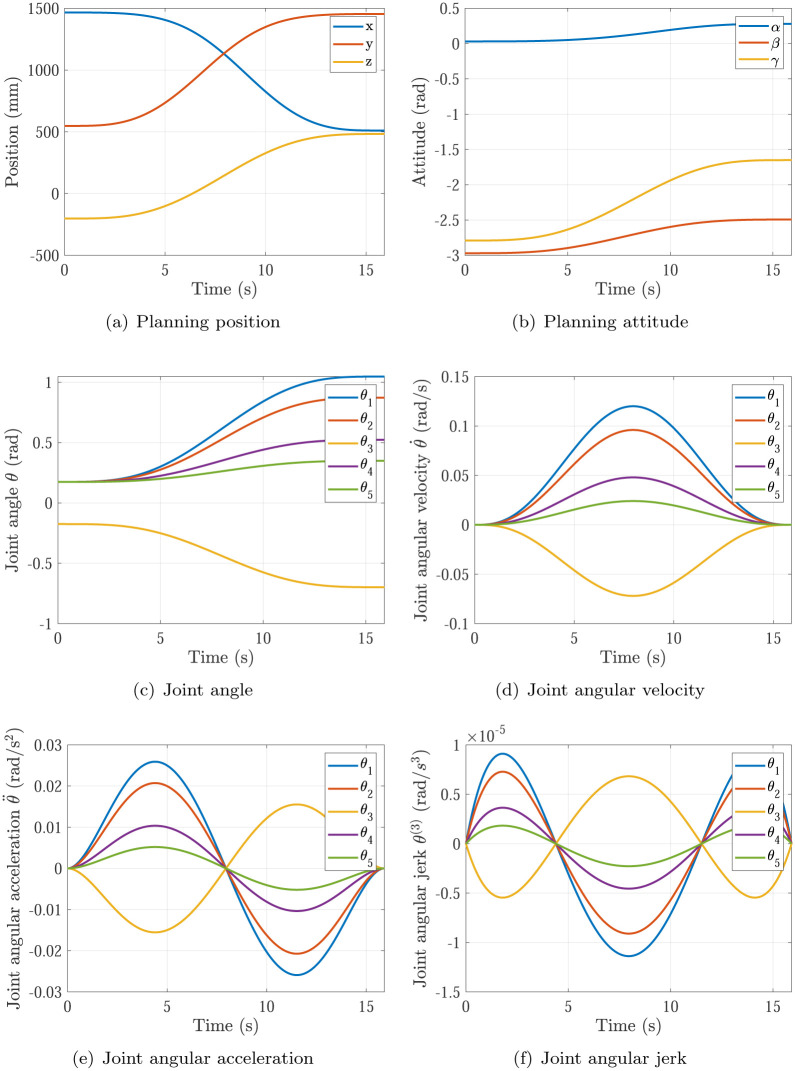
The trajectory planning results of Simulation 5 based on the proposed optimization method. (a) Planning position, (b) Planning attitude, (c) Joint angle, (d) Joint angular velocity, (e) Joint angular acceleration, (f) Joint angular jerk.

**Fig 10:**
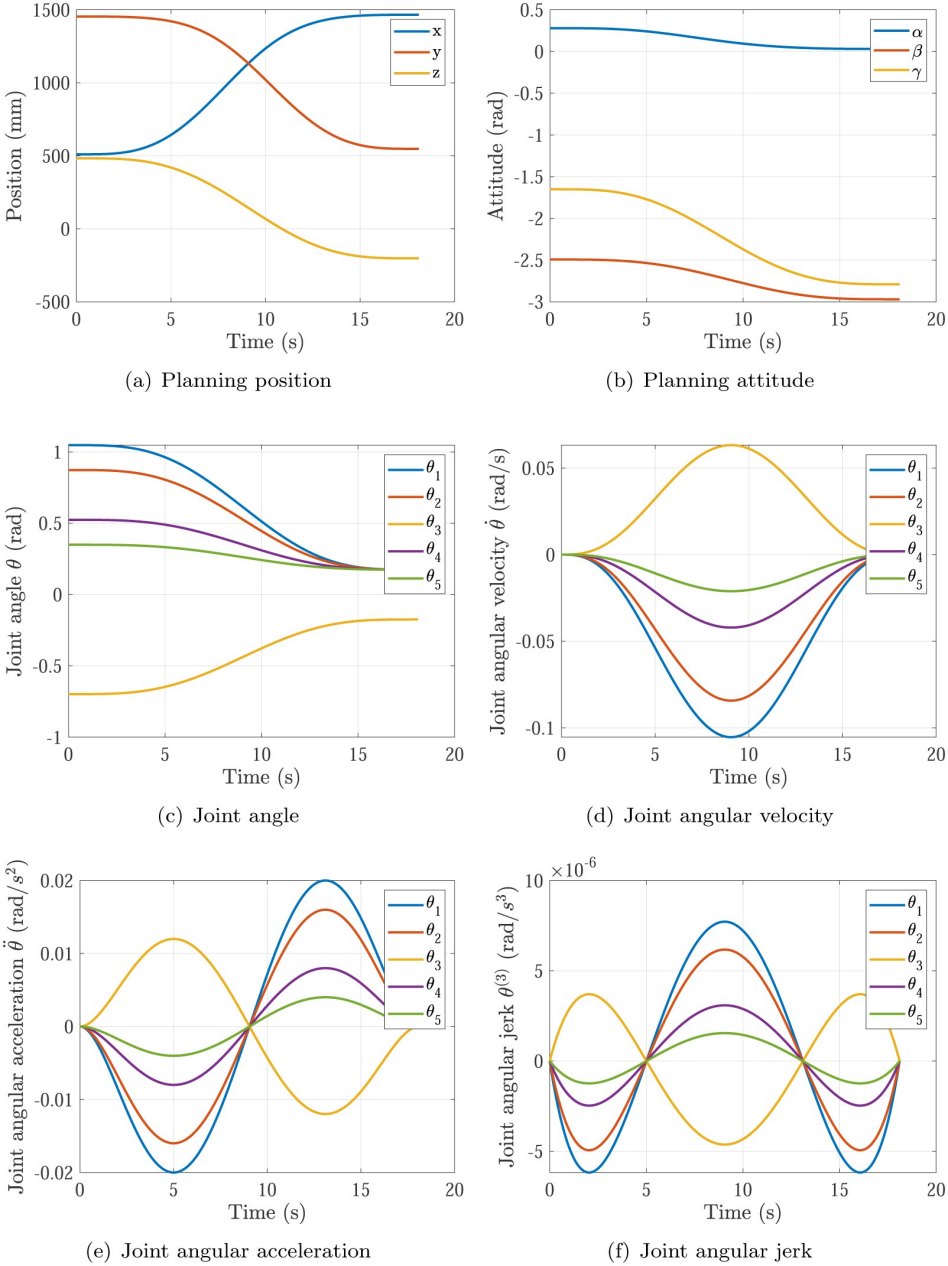
The trajectory planning results of Simulation 6 based on the proposed optimization method. (a) Planning position, (b) Planning attitude, (c) Joint angle, (d) Joint angular velocity, (e) Joint angular acceleration, (f) Joint angular jerk.

**Table 4:** Comparisons of simulations.

Simulations	$\text{max}\;\{|\dot{\theta }|\}$	$\text{max}\;\{|\ddot{\theta }|\}$	*T* = *t*_f_ − *t*_0_	$\dddot{\theta }$
Simulation 1	0.1636rad/s	0.0504rad/s^2^	10s	non-continuous
Simulation 2	0.0818rad/s	0.0126rad/s^2^	20s	non-continuous
Simulation 3	0.1909rad/s	0.0656rad/s^2^	10s	continuous
Simulation 4	0.0954rad/s	0.0164rad/s^2^	20s	continuous
Simulation 5	0.1200rad/s	0.0259rad/s^2^	15.908s	continuous
Simulation 6	0.1054rad/s	0.0200rad/s^2^	18.106s	continuous

Comparing Simulations 1, 2 & 5, it can be found that the maximums of joint angular accelerations are all inside the required angular acceleration of actuators specifications ${\ddot{\theta }}_{\text{max}}=0.08{\text{rad}/\text{s}}^{2}$. It means the P control of $k_{\ddot{\theta }}$ in Eq (6) does not work in the three simulations. In Simulation 1, the maximum of joint angular velocity $\text{max}\;\{|\dot{\theta }|\}=0.1636\text{rad}/\text{s}$ is larger than the required angular velocity of actuators specifications ${\dot{\theta }}_{\text{max}}=0.12\text{rad}/\text{s}$. In Simulation 2, the maximum of joint angular velocity $\text{max}\;\{|\dot{\theta }|\}=0.0818\text{rad}/\text{s}$ is smaller than the required angular velocity of actuators specifications ${\dot{\theta }}_{\text{max}}=0.12\text{rad}/\text{s}$. In Simulation 5 based on the proposed optimization method, the maximum of joint angular velocity $\text{max}\;\{|\dot{\theta }|\}=0.12\text{rad}/\text{s}$ just equal to the required angular velocity of actuators specifications ${\dot{\theta }}_{\text{max}}=0.12\text{rad}/\text{s}$. That means the planning time should be increased in Simulation 1 and decreased in Simulation 2 to make time optimal and make actuators specifications satisfied, which is achieved by the time optimal controller Eq (6). Noting that the main effector is the maximum of joint angular velocity $\text{max}\;\{|\dot{\theta }|\}$.

Comparing Simulations 3, 4 & 6, it can be found that the maximum of joint angular velocity in Simulation 4 $\text{max}\;\{|\dot{\theta }|\}=0.0954\text{rad}/\text{s}$ is inside the required angular velocity of actuators specifications ${\dot{\theta }}_{\text{max}}=0.12\text{rad}/\text{s}$, while the maximum of joint angular velocity in Simulation 3 $\text{max}\;\{|\dot{\theta }|\}=0.1909\text{rad}/\text{s}$ is not. It means the P control of $k_{\dot{\theta }}$ in Eq (6) does not work in Simulations 4, and the P control of $k_{\dot{\theta }}$ in Eq (6) does work at the start of optimizations and does not work at the end of optimizations in Simulation 3, and only the P control of $k_{\ddot{\theta }}$ in Eq (6) does work at the end of optimizations in Simulation 3. In Simulation 3, the maximum of joint angular acceleration $\text{max}\;\{|\ddot{\theta }|\}=0.0656{\text{rad}/\text{s}}^{2}$ is larger than the required angular acceleration of actuators specifications ${\ddot{\theta }}_{\text{max}}=0.02{\text{rad}/\text{s}}^{2}$. In Simulation 4, the maximum of joint angular acceleration $\text{max}\;\{|\ddot{\theta }|\}=0.0164{\text{rad}/\text{s}}^{2}$ is smaller than the required angular acceleration of actuators specifications ${\ddot{\theta }}_{\text{max}}=0.02{\text{rad}/\text{s}}^{2}$. In Simulation 6 based on the proposed optimization method, the maximum of joint angular acceleration $\text{max}\;\{|\ddot{\theta }|\}=0.02\text{rad}/\text{s}$ just equal to the required angular acceleration of actuators specifications ${\ddot{\theta }}_{\text{max}}=0.02{\text{rad}/\text{s}}^{2}$. That means the planning time should be increased in Simulation 3 and decreased in Simulation 4 to make time optimal and make actuators specifications satisfied, which is achieved by the time optimal controller Eq (6) as well. Noting that the main effector is the maximum of joint angular acceleration $\text{max}\;\{|\ddot{\theta }|\}$.

Comparing Simulations 1 & 2 with 3, 4, 5 & 6, it can be found that the trajectory planning based on seventh order polynomial interpolation can guarantee the planning jerk smooth and stable [37], but the trajectory planning based on fifth order polynomial interpolation cannot. Both of them can generate the continuous acceleration profile. Comparing Simulations 1 & 3 and Comparing Simulations 2 & 4, it can be found that the trajectory planning based on seventh order polynomial interpolation has higher maximum of joint angular velocity $\text{max}\;\{|\dot{\theta }|\}$ and higher maximum of joint angular acceleration $\text{max}\;\{|\ddot{\theta }|\}$ than that of the trajectory planning based on fifth order polynomial interpolation. That’s the trade-off.

**Fig 11:**
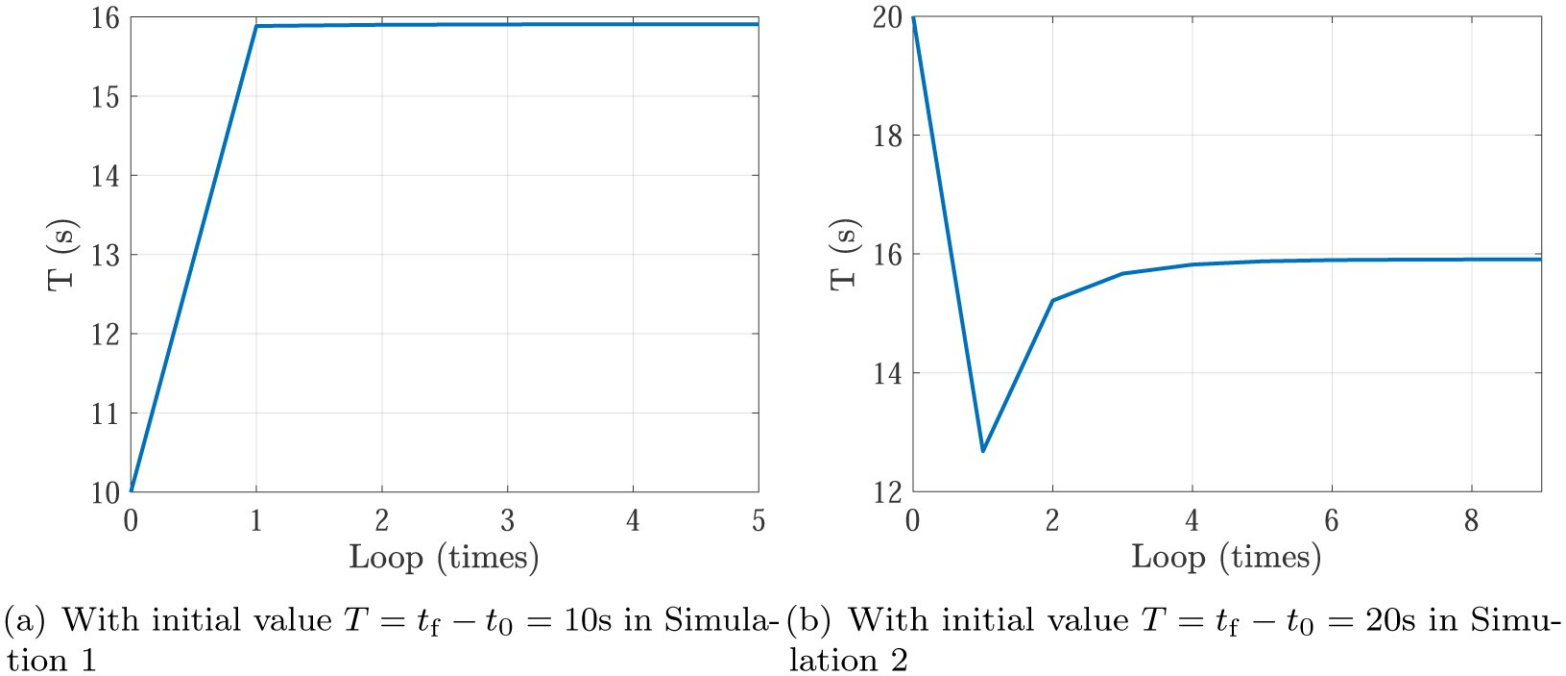
The optimization process of planning time *T* = *t*_f_ − *t*_0_ in Simulation 5. (a) With initial value *T* = *t*_f_ − *t*_0_ = 10s in Simulation 1, (b) With initial value *T* = *t*_f_ − *t*_0_ = 20s in Simulation 2.

**Fig 12:**
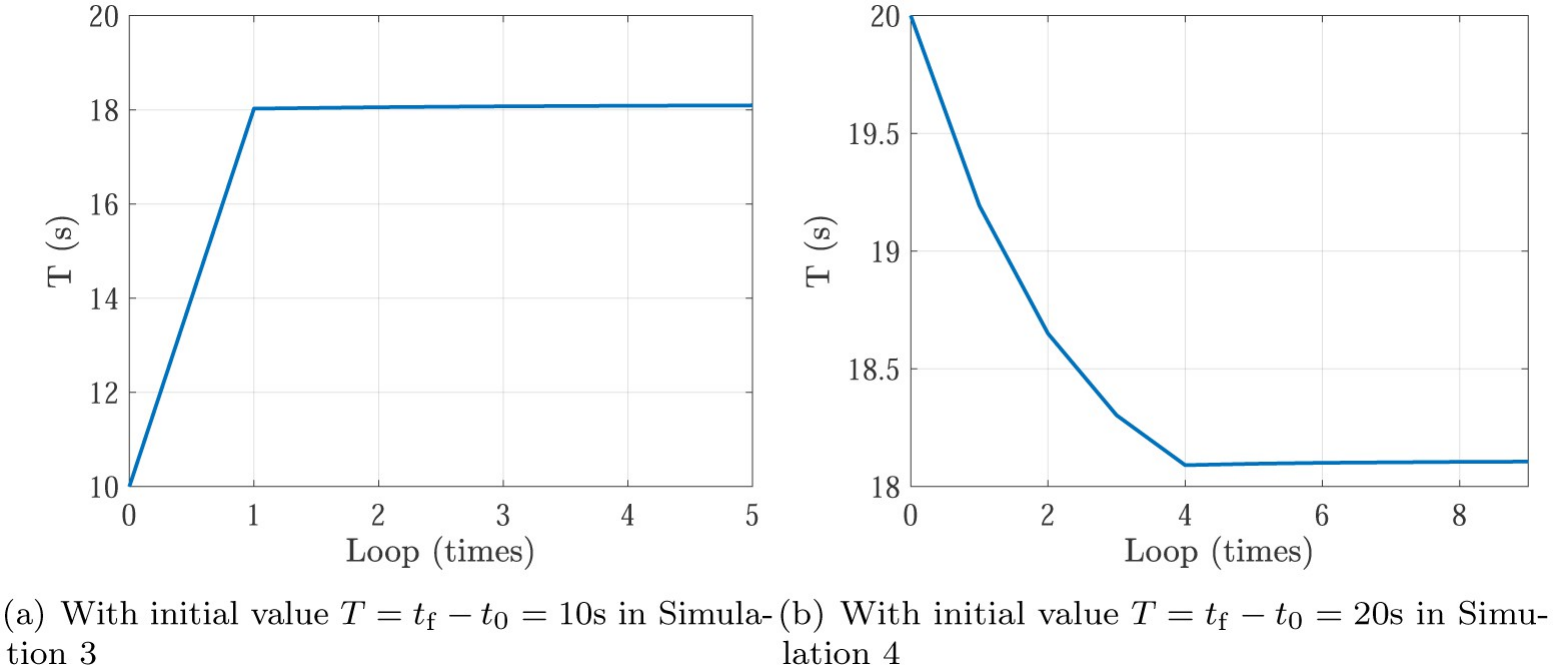
The optimization process of planning time *T* = *t*_f_ − *t*_0_ in Simulation 6. (a) With initial value *T* = *t*_f_ − *t*_0_ = 10s in Simulation 3, (b) With initial value *T* = *t*_f_ − *t*_0_ = 20s in Simulation 4.

Simulation 5 is the optimization results of Simulation 1 & 2, and Simulation 6 is the optimization results of Simulation 3 & 4. In Simulation 5, the optimization process of planning time *T* = *t*_f_ − *t*_0_ with initial value in Simulation 1 (*T* = *t*_f_ − *t*_0_ = 10s) and Simulation 2 (*T* = *t*_f_ − *t*_0_ = 20s) are shown in Fig 11. In Simulation 6, the optimization process of planning time *T* = *t*_f_ − *t*_0_ with initial value in Simulation 3 (*T* = *t*_f_ − *t*_0_ = 10s) and Simulation 4 (*T* = *t*_f_ − *t*_0_ = 20s) are shown in Fig 12. Actually, in Figs 11(b) and 12(b), the decreasing curve represents Case 1 and the planning time should be decreased to close to the optimal planning time, while the increasing curve in Figs 11 and 12 represents Case 2 and the planning time should be increased to close to the optimal planning time. Finally, the planning time is optimized as 15.908s in Simulation 5 and 18.106s in Simulation 6. Meanwhile, the actuators specifications are satisfied. It can be shown that, the optimizing loops are less than 10, which shows the trajectory optimization can be finished quickly. Noting that there exists an overshoot in the decreasing curve. The phenomenon is similar to P control in PID control.

**Fig 13:**
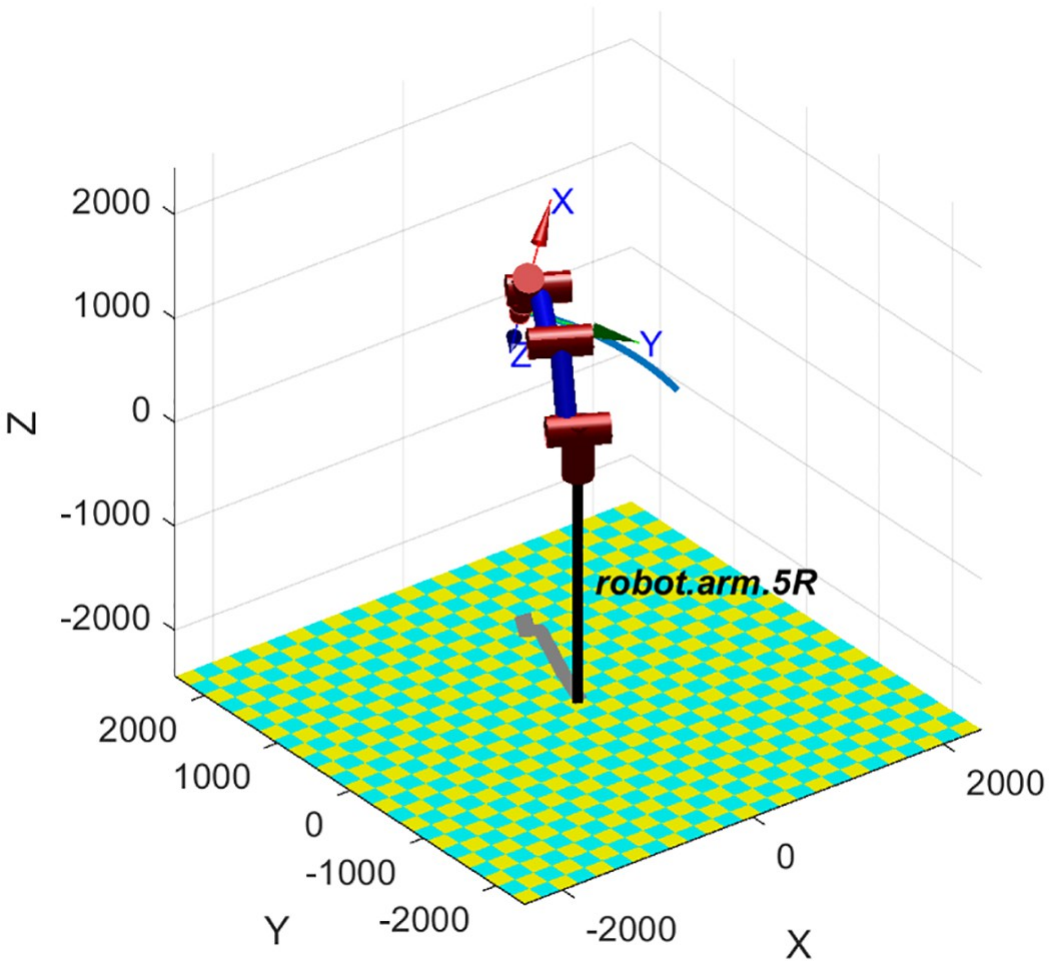
The planning trajectory based on the proposed optimization method in Simulation 5.

To evaluate the performance of calculation efficiency of proposed optimization method, comparative simulations based on Simulation 5 are implemented with two iterative algorithms: GA [15] and PSO [17], and one hierarchical three-layer trajectory planning framework [29]. Compared with these two algorithms, the required time for trajectory optimization based on the proposed optimization method is much lower, which is about 19ms, while the GA and PSO algorithms would cost about 1326ms and 1764ms, respectively, and the hierarchical three-layer trajectory planning framework would cost about 42ms. Noting that the required time is the calculated time of PC to get the optimized trajectory, instead of the planning time. And, the proposed method combining event-trigger and conditional P control, GA, PSO and the hierarchical three-layer trajectory planning framework are employed to achieve the time-optimal trajectory planning individually. Therefore, the proposed optimization method owns higher efficiency of trajectory optimization. The planning trajectory based on the proposed optimization method in Simulation 5 can be drawn by Robotics Toolbox in Matlab, as shown in Fig 13, where the blue curve is the planning trajectory.

### 4.2 Experiments

The experimental platform, a manipulator, is shown in Fig 2a, and its system parameters are the same as simulation parameters Table 2. The maximum velocity and acceleration of actuators specifications of each joint of manipulator are shown in Table 5. The proposed optimization method is employed to do the trajectory planning: repeating the point to point trajectory *p*_A_ → *p*_B_ → *p*_C_ → *p*_D_ → *p*_C_ → *p*_B_ → *p*_A_. The trajectory planning can be divided into three point to point movements: *p*_A_ ↔ *p*_B_, *p*_B_ ↔ *p*_C_, *p*_C_ ↔ *p*_D_.

**Table 5:** Simulation parameters.

Parameter	Value
$p_{\text{A}}=\left[\begin{array}{c} x_{\text{A}}\\ y_{\text{A}}\\ z_{\text{A}}\\ \alpha_{\text{A}}\\ \beta_{\text{A}}\\ \gamma_{\text{A}} \end{array}\right]$	$\left[\begin{array}{c} 0\text{mm}\\ -600\text{mm}\\ -200\text{mm}\\ 0\text{rad}\\ -\pi \text{rad}\\ 0\text{rad} \end{array}\right]$
$p_{\text{B}}=\left[\begin{array}{c} x_{\text{B}}\\ y_{\text{B}}\\ z_{\text{B}}\\ \alpha_{\text{B}}\\ \beta_{\text{B}}\\ \gamma_{\text{B}} \end{array}\right]$	$\left[\begin{array}{c} 0\text{mm}\\ -600\text{mm}\\ 0\text{mm}\\ 0\text{rad}\\ -\pi \text{rad}\\ 0\text{rad} \end{array}\right]$
$p_{\text{C}}=\left[\begin{array}{c} x_{\text{C}}\\ y_{\text{C}}\\ z_{\text{C}}\\ \alpha_{\text{C}}\\ \beta_{\text{C}}\\ \gamma_{\text{C}} \end{array}\right]$	$\left[\begin{array}{c} 0\text{mm}\\ -600\text{mm}\\ 0\text{mm}\\ 0\text{rad}\\ -\pi \text{rad}\\ 0\text{rad} \end{array}\right]$
$p_{\text{D}}=\left[\begin{array}{c} x_{\text{D}}\\ y_{\text{D}}\\ z_{\text{D}}\\ \alpha_{\text{D}}\\ \beta_{\text{D}}\\ \gamma_{\text{D}} \end{array}\right]$	$\left[\begin{array}{c} 0\text{mm}\\ -600\text{mm}\\ 0\text{mm}\\ 0\text{rad}\\ -\pi \text{rad}\\ 0\text{rad} \end{array}\right]$
$\left[\begin{array}{c} {\dot{\theta }}_{\text{max}}\\ {\ddot{\theta }}_{\text{max}}\\ k_{\dot{\theta }}\\ k_{\ddot{\theta }} \end{array}\right]$	$\left[\begin{array}{c} 12\text{rad}/\text{s}\\ 20{\text{rad}/\text{s}}^{2}\\ 0.12\\ 0.02 \end{array}\right]$
*ε*_*θ*_	0.001rad
Initial $\left[\begin{array}{c} T=t_{\text{f}}\\ t_{0} \end{array}\right]$	*p*_A_ → *p*_B_, *p*_B_ → *p*_C_, *p*_C_ → *p*_D_ all are $\left[\begin{array}{c} 3\text{s}\\ 0 \end{array}\right]$
Optimal $\left[\begin{array}{c} T=t_{\text{f}}\\ t_{0} \end{array}\right]$	$p_{\text{A}}\to p_{\text{B}},p_{\text{C}}\to p_{\text{D}}:\left[\begin{array}{c} 1.537\text{s}\\ 0 \end{array}\right],p_{\text{B}}\to p_{\text{C}}:\left[\begin{array}{c} 1.494\text{s}\\ 0 \end{array}\right]$
$\text{max}\;\{|\dot{\theta }|\}$	*p*_A_ → *p*_B_, *p*_C_ → *p*_D_: 8.9423rad/s, p_B_ → p_C_: 8.6884rad/s
$\text{max}\;\{|\ddot{\theta }|\}$	*p*_A_ → *p*_B_, *p*_B_ → *p*_C_, *p*_C_ → *p*_D_ all are 20rad/s^2^

**Fig 14:**
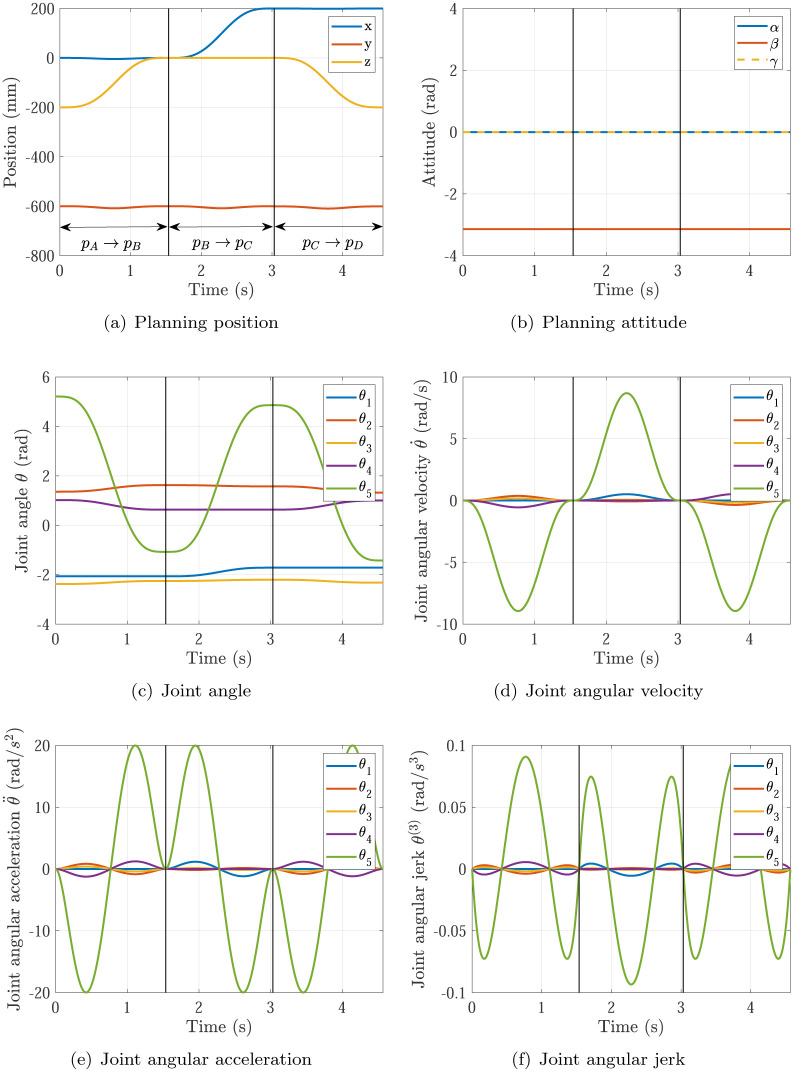
The trajectory planning results of experiment based on the proposed optimization method. (a) Planning position, (b) Planning attitude, (c) Joint angle, (d) Joint angular velocity, (e) Joint angular acceleration, (f) Joint angular jerk.

The trajectory planning results of experiment based on the proposed optimization method is shown in Fig 14. Some of experimental results are also shown in Table 5. In the initial panning time *T* = *t*_f_ = 3s of every point to point movement without optimization, the maximum values of planning velocity and acceleration should be both smaller than the maximum velocity and acceleration of actuators specifications in each joints (Event 1), which means the panning time *T* = *t*_f_ = 3s can be smaller to make the manipulator move faster and the actuators specifications can be satisfied simultaneously. In the optimal panning time with the proposed optimization method, the optimized planning time of *p*_A_ → *p*_B_, *p*_B_ → *p*_C_, *p*_C_ → *p*_D_ are 1.537s, 1.494s, 1.537s, respectively. And the maximums of joint angular velocities $\text{max}\;\{|\dot{\theta }|\}=8.9342\text{rad}/\text{s}$ are all inside the required angular velocities of actuators specifications ${\ddot{\theta }}_{\text{max}}=12\text{rad}/\text{s}$ (see Fig 14(d)), while the maximums of joint angular accelerations $\text{max}\;\{|\dot{\theta }|\}=20{\text{rad}/\text{s}}^{2}$ just equal to the required angular accelerations of actuators specifications ${\dot{\theta }}_{\text{max}}=20{\text{rad}/\text{s}}^{2}$ (see Fig 14(e)). As thus, the planning times of three point to point movements all reach their optimal values.

**Fig 15:**
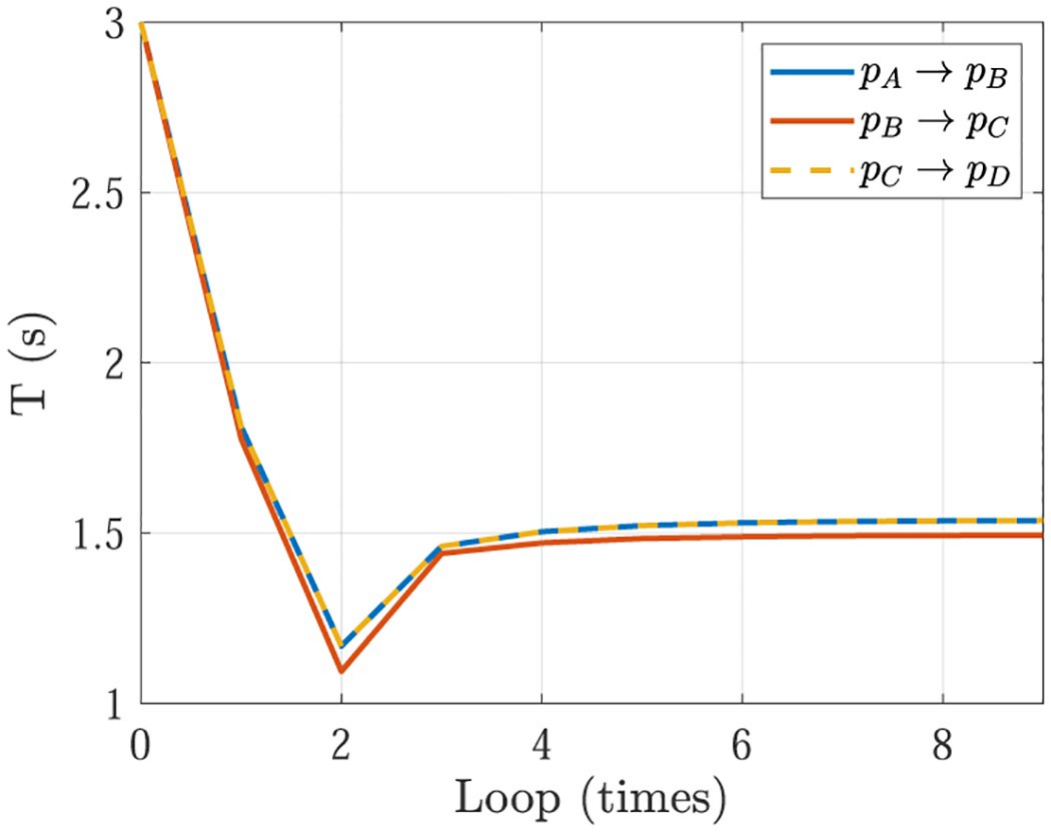
The optimization process of planning time *T* = *t*_f_ − *t*_0_ in experiment.

The optimization process of planning time *T* = *t*_f_ − *t*_0_ with initial value 3s of three point to point movements are shown in Fig 15. The decreasing curve represents Case 1 and the planning time should be decreased to close to the optimal planning time, while the increasing curve represents Case 2 and the planning time should be increased to close to the optimal planning time. Finally, the planning times of three movements of point to point are respectively optimized as 1.537s, 1.494s, 1.537s. Meanwhile, the actuators specifications are satisfied. It can be shown that, the optimizing loops are less than 10, which shows the trajectory optimization can be finished quickly. It makes room for the applications of proposed trajectory optimization on the micro controller with low computing capability and high real-time performance requirement.

## 5 Conclusions

A method of time-optimal trajectory planning based on polynomial interpolation, event-trigger mechanism and conditional P control is proposed in this paper. The main contributions can be concluded as follows:

- The forward kinematics and inverse kinematics of a five axis manipulator are deduced. And, a simple method to choose one appropriate solution from multi solutions of inverse kinematics is proposed.
- Based on seventh order polynomial interpolation, event-trigger mechanism and conditional P control, an easy-implemented optimization method of trajectory planning is proposed to guarantee that the obtained trajectory is time optimal and satisfies actuators specifications.
- The effectiveness and efficiency of proposed optimization method are validated by comparative simulations and experiments.

Future work will focus on easy-implemented optimization method of trajectory planning with more constraints and objectives.

## 6 Appendix

## A Forward kinematics

The transformation matrix from coordinate system {i-1} (*o*_*i*−1_*x*_*i*−1_*y*_*i*−1_*z*_*i*−1_) to coordinate system {i} (*o*_*i*_*x*_*i*_*y*_*i*_*z*_*i*_) can be written as

$$\begin{array}{c} \begin{array}{c} A_{i}^{i-1}=R\left({z_{i}}_{-1},\theta_{i}\right)Trans\left(0,0,d_{i}\right)Trans\left(a_{i},0,0\right)R\left(x_{i},\alpha_{i}\right)\\ =R\left(z_{i-1}\theta_{i}\right)Trans\left(a_{i},0,d_{i}\right)R\left(x_{i},\alpha_{i}\right)\\ =\left[\begin{array}{cccc} \text{cos}\;\theta_{i} & -\text{sin}\;\theta_{i} & 0 & 0\\ \text{sin}\;\theta_{i} & \text{cos}\;\theta_{i} & 0 & 0\\ 0 & 0 & 1 & 0\\ 0 & 0 & 0 & 1 \end{array}\right]\left[\begin{array}{cccc} 1 & 0 & 0 & a_{i}\\ 0 & 1 & 0 & 0\\ 0 & 0 & 1 & d_{i}\\ 0 & 0 & 0 & 1 \end{array}\right]\\ \left[\begin{array}{cccc} 1 & 0 & 0 & 0\\ 0 & \text{cos}\;\alpha_{i} & -\text{sin}\;\alpha_{i} & 0\\ 0 & \text{sin}\;\alpha_{i} & \text{cos}\;\alpha_{i} & 0\\ 0 & 0 & 0 & 1 \end{array}\right]\\ =\left[\begin{array}{cccc} \text{cos}\;\theta_{i} & -\text{cos}\;\alpha_{i}\;\text{sin}\;\theta_{i} & \text{sin}\;\alpha_{i}\;\text{sin}\;\theta_{i} & a_{i}\;\text{cos}\;\theta_{i}\\ \text{sin}\;\theta_{i} & \text{cos}\;\alpha_{i}\;\text{cos}\;\theta_{i} & -\text{sin}\;\alpha_{i}\;\text{cos}\;\theta_{i} & a_{i}\;\text{sin}\;\theta_{i}\\ 0 & \text{sin}\;\alpha_{i} & \text{cos}\;\alpha_{i} & d_{i}\\ 0 & 0 & 0 & 1 \end{array}\right] \end{array} \end{array}$$ (7)

Substituting the DH parameters into the transformation matrix Eq (7), the homogeneous transformation matrix between each coordinate system can be obtained as

$$\begin{array}{c} \begin{array}{c} A_{1}^{0}=\left[\begin{array}{cccc} \text{cos}\;\theta_{1} & 0 & \text{sin}\;\theta_{1} & 0\\ \text{sin}\;\theta_{1} & 0 & -\text{cos}\;\theta_{1} & 0\\ 0 & 1 & 0 & d_{1}\\ 0 & 0 & 0 & 1 \end{array}\right]\\ A_{2}^{1}=\left[\begin{array}{cccc} \text{cos}\;\theta_{2} & -\text{sin}\;\theta_{2} & 0 & a_{2}\;\text{cos}\;\theta_{2}\\ \text{sin}\;\theta_{2} & \text{cos}\;\theta_{2} & 0 & a_{2}\;\text{sin}\;\theta_{2}\\ 0 & 0 & 1 & d_{2}\\ 0 & 0 & 0 & 1 \end{array}\right]\\ A_{3}^{2}=\left[\begin{array}{cccc} \text{cos}\;\theta_{3} & -\text{sin}\;\theta_{3} & 0 & a_{3}\;\text{cos}\;\theta_{3}\\ \text{sin}\;\theta_{3} & \text{cos}\;\theta_{3} & 0 & a_{3}\;\text{sin}\;\theta_{3}\\ 0 & 0 & 1 & d_{3}\\ 0 & 0 & 0 & 1 \end{array}\right]\\ A_{4}^{3}=\left[\begin{array}{cccc} \text{cos}\;\theta_{4} & 0 & -\text{sin}\;\theta_{4} & 0\\ \text{sin}\;\theta_{4} & 0 & \text{cos}\;\theta_{4} & 0\\ 0 & -1 & 0 & d_{4}\\ 0 & 0 & 0 & 1 \end{array}\right]\\ A_{5}^{4}=\left[\begin{array}{cccc} \text{cos}\;\theta_{5} & -\text{sin}\;\theta_{5} & 0 & 0\\ \text{sin}\;\theta_{5} & \text{cos}\;\theta_{5} & 0 & 0\\ 0 & 0 & 1 & d_{5}\\ 0 & 0 & 0 & 1 \end{array}\right] \end{array} \end{array}$$ (8)

After obtaining the homogeneous transformation matrix between coordinate system, the homogeneous transformation matrix between the coordinate system of end effector {5} and the base coordinate system {0} can be obtained through the chain rule as

$$\begin{array}{c} A_{5}^{0}=A_{1}^{0}A_{2}^{1}A_{3}^{2}A_{4}^{3}A_{5}^{4}=\left[\begin{array}{cccc} n_{x} & o_{x} & a_{x} & p_{x}\\ n_{y} & o_{y} & a_{y} & p_{y}\\ n_{z} & o_{z} & a_{z} & p_{z}\\ 0 & 0 & 0 & 1 \end{array}\right] \end{array}$$ (9)

where,

$$\begin{array}{c} \left\{ \begin{array}{c} n_{x}=\text{cos}\;\theta_{1}\text{cos}\left(\theta_{2}+\theta_{3}+\theta_{4}\right)\text{cos}\;\theta_{5}-\text{sin}\;\theta_{1}\;\text{sin}\;\theta_{5}\\ o_{x}=-\text{cos}\;\theta_{1}\text{cos}\left(\theta_{2}+\theta_{3}+\theta_{4}\right)\text{sin}\;\theta_{5}-\text{cos}\;\theta_{5}\;\text{sin}\;\theta_{1}\\ a_{x}=-\text{cos}\;\theta_{1}\text{sin}\left(\theta_{2}+\theta_{3}+\theta_{4}\right) \end{array},\right.\\ \left\{ \begin{array}{c} n_{y}=\text{cos}\left(\theta_{2}+\theta_{3}+\theta_{4}\right)\text{cos}\;\theta_{5}\;\text{sin}\;\theta_{1}+\text{cos}\;\theta_{1}\;\text{sin}\;\theta_{5}\\ o_{y}=\text{cos}\;\theta_{1}\;\text{cos}\;\theta_{5}-\text{cos}\left(\theta_{2}+\theta_{3}+\theta_{4}\right)\text{sin}\;\theta_{1}\;\text{sin}\;\theta_{5}\\ a_{y}=-\text{sin}\;\theta_{1}\text{sin}\left(\theta_{2}+\theta_{3}+\theta_{4}\right) \end{array},\right.\\ \left\{ \begin{array}{c} n_{z}=\text{cos}\;\theta_{5}\text{sin}\left(\theta_{2}+\theta_{3}+\theta_{4}\right)\\ o_{z}=-\text{sin}\left(\theta_{2}+\theta_{3}+\theta_{4}\right)\text{sin}\;\theta_{5}\\ a_{z}=\text{cos}\left(\theta_{2}+\theta_{3}+\theta_{4}\right) \end{array},\right.\\ \left\{ \begin{array}{c} p_{x}=\text{sin}\;\theta_{1}\left(d_{2}+d_{3}+d_{4}\right)+\text{cos}\;\theta_{1}\left(a_{2}\;\text{cos}\;\theta_{2}+a_{3}\text{cos}\left(\theta_{2}+\theta_{3}\right)-d_{5}\text{sin}\left(\theta_{2}+\theta_{3}+\theta_{4}\right)\right)\\ p_{y}=-\text{cos}\;\theta_{1}\left(d_{2}+d_{3}+d_{4}\right)+\text{sin}\;\theta_{1}\left(a_{2}\;\text{cos}\;\theta_{2}+a_{3}\text{cos}\left(\theta_{2}+\theta_{3}\right)-d_{5}\text{sin}\left(\theta_{2}+\theta_{3}+\theta_{4}\right)\right)\\ p_{z}=d_{1}+a_{3}\text{sin}\left(\theta_{2}+\theta_{3}\right)+a_{2}\text{sin}\left(\theta_{2}\right)+d_{5}\text{cos}\left(\theta_{2}+\theta_{3}+\theta_{4}\right) \end{array},\right. \end{array}$$

Set the coordinate system {0} in the first axis as the base/reference coordinate system of manipulator, then the pose transformation matrix from the end effector to the base is shown in (9). In the forward kinematics, the position parameters *x*, *y*, *z* and attitude parameters *α*, *β*, *γ* of the end effector are dependent variables, while the joint angles *θ*_*i*_(*i* = 1, 2, 3, 4, 5) are independent variables. According to the chain rule, the forward kinematics and its analytical solutions of the manipulator can be directly obtained as shown in (9), where ${\left[\begin{array}{ccc} p_{x} & p_{y} & p_{z} \end{array}\right]}^{\text{T}}$ are the parameters *x*, *y*, *z* of end effector of manipulator on the base coordinate system {0}, that is

$$\begin{array}{c} \left\{ \begin{array}{c} x=p_{x}\\ y=p_{y}\\ z=p_{z} \end{array}\right. \end{array}$$ (10)

${\left[\begin{array}{ccc} n_{x} & n_{y} & n_{z} \end{array}\right]}^{\text{T}},{\left[\begin{array}{ccc} o_{x} & o_{y} & o_{z} \end{array}\right]}^{\text{T}},{\left[\begin{array}{ccc} a_{x} & a_{y} & a_{z} \end{array}\right]}^{\text{T}}$ are the attitude related parameters of end effector of manipulator on the base coordinate system {0}. Based on the rotation order: Z-Y-X, the attitude parameters (Euler angles) *α*, *β*, *γ* can be obtained as

$$\begin{array}{c} \left\{ \begin{array}{c} \alpha =\text{arctan}2(o_{z},a_{z})\\ \beta =\text{arctan}2(-n_{z},\sqrt{n_{z}^{2}+a_{z}^{2}})\\ \gamma =\text{arctan}2(n_{y},n_{x}) \end{array}\right. \end{array}$$ (11)

where arctan2(*Y*, *X*) is a C function, which returns the arctangent of *Y*/*X* in radians. The sign of the values of *Y* and *X* determines the correct quadrant.

## B Inverse kinematics

In the inverse kinematics, the joint angles *θ*_*i*_(*i* = 1, 2, 3, 4, 5) are dependent variables, while the position parameters *x*, *y*, *z* and attitude parameters *α*, *β*, *γ* of end effector of manipulator are independent variables. Because the end effector of 5 axis manipulator has only 5 DoFs in Cartesian space, only 5 parameters of manipulator (3 position parameters and 2 attitude parameters) can be determined by given 5 joint angles. And the last one attitude parameter is not an independent variable, which can be calculated via the forward kinematics Eqs (8)–(11).

Set the five parameters of manipulator: position parameters *p*_*x*_, *p*_*y*_, *p*_*z*_, the rotation angle *α* around *x*_0_ axis and *β* around *y*_0_ axis in the base coordinate system {0} can be determined by joint angles *θ*_*i*_(*i* = 1, 2, 3, 4, 5), then the rotation angle *γ* around *z*_0_ axis in the base coordinate system {0} is the non-independent variable, which can be calculated via the forward kinematics Eqs (8)–(11). Thus, given five parameters $\left[\begin{array}{ccccc} p_{x} & p_{y} & p_{z} & \alpha & \beta \end{array}\right]$ of manipulator, the five joint angles *θ*_*i*_(*i* = 1, 2, 3, 4, 5) can be deduced as follows.

Rewriting Eq (9) yields

$$\begin{array}{c} {\left(A_{1}^{0}\right)}^{-1}A_{5}^{0}=A_{2}^{1}A_{3}^{2}A_{4}^{3}A_{5}^{4} \end{array}$$ (12)

If the first column of matrix Eq (12) is equal, the following equations can be obtained

$$\begin{array}{c} n_{x}\;\text{cos}\;\theta_{1}+n_{y}\;\text{sin}\;\theta_{1}=\text{cos}\;\theta_{234}\;\text{cos}\;\theta_{5} \end{array}$$ (13)

$$\begin{array}{c} -n_{z}=\text{cos}\;\theta_{5}\;\text{sin}\;\theta_{234} \end{array}$$ (14)

$$\begin{array}{c} n_{x}\;\text{sin}\;\theta_{1}-n_{y}\;\text{cos}\;\theta_{1}=\text{sin}\;\theta_{5} \end{array}$$ (15)

where *θ*_234_ = *θ*_2_ + *θ*_3_ + *θ*_4_.

If the fourth column of matrix Eq (12) is equal, the following equations can be obtained

$$\begin{array}{c} a_{3}\;\text{cos}\;\theta_{23}+a_{2}\;\text{cos}\;\theta_{2}-d_{5}\;\text{sin}\;\theta_{234}=p_{x}\;\text{cos}\;\theta_{1}+p_{y}\;\text{sin}\;\theta_{1} \end{array}$$ (16)

$$\begin{array}{c} a_{3}\;\text{sin}\;\theta_{23}+d_{5}\;\text{cos}\;\theta_{234}+a_{2}\;\text{sin}\;\theta_{2}=p_{z}-d_{1} \end{array}$$ (17)

$$\begin{array}{c} d_{2}+d_{3}+d_{4}=p_{x}\;\text{sin}\;\theta_{1}-p_{y}\;\text{cos}\;\theta_{1} \end{array}$$ (18)

where *θ*_23_ = *θ*_2_ + *θ*_3_.

The first joint angle *θ*_1_ can be obtained via Eq (18). And there exists two solutions, which are respectively

$$\begin{array}{c} \theta_{1}=2\;\text{arctan}\left[\frac{p_{x}\pm \frac{1}{2}\sqrt{4p_{x}^{2}-4(d_{2}+d_{3}+d_{4}-p_{y})(d_{2}+d_{3}+d_{4}+p_{y})}}{d_{2}+d_{3}+d_{4}-p_{y}}\right] \end{array}$$ (19)

Combining Eq (19) with Eq (15), the joint angle *θ*_5_ can be obtained. And there also exists two solutions, which are respectively

$$\begin{array}{c} \begin{array}{c} \theta_{5}=-\text{arcsin}\left(ny\;\text{cos}\;\theta_{1}-nx\;\text{sin}\;\theta_{1}\right)\\ \theta_{5}=\pi +\text{arcsin}\left(ny\;\text{cos}\;\theta_{1}-nx\;\text{sin}\;\theta_{1}\right) \end{array} \end{array}$$ (20)

Combining the calculated joint angle *θ*_1_ in Eq (19), *θ*_5_ in Eq (20) with Eqs (13) and (14), the unique solution of *θ*_234_ can be obtained as

$$\begin{array}{c} \theta_{234}=a\;\text{tan}\;2\left(n_{z}/\text{cos}\;\theta_{5},\left(n_{x}\;\text{cos}\;\theta_{1}+n_{y}\;\text{sin}\;\theta_{1}\right)/\text{cos}\;\theta_{5}\right) \end{array}$$ (21)

Based on Eqs (19)–(21), the joint angles *θ*_2_, *θ*_3_ can be obtained via Eqs (16) and (17). Eqs (16) and (17) can be rewritten as

$$\begin{array}{c} \begin{array}{c} a_{2}\;\text{cos}\;\theta_{2}+a_{3}\;\text{cos}\;\theta_{23}=k_{1}\\ a_{2}\;\text{sin}\;\theta_{2}+a_{3}\;\text{sin}\;\theta_{23}=k_{2} \end{array} \end{array}$$ (22)

where *k*_1_ = *p*_*x*_ cos *θ*_1_ + *p*_*y*_ sin *θ*_1_ + *d*_5_ sin *θ*_234_, *k*_2_ = *p*_*z*_ − *d*_1_ − *d*_5_ cos *θ*_234_, and *k*_1_, *k*_2_ are known.

Then the joint angle *θ*_3_ can be obtained as

$$\begin{array}{c} \theta_{3}=\pm a\;\text{cos}\left(\left(k_{1}^{2}+k_{2}^{2}-{a_{2}}^{2}-{a_{3}}^{2}\right)/\left(2a_{2}a_{3}\right)\right) \end{array}$$ (23)

By solving the sum of squares of Eq (22), the joint angle *θ*_2_ can be obtained as

$$\begin{array}{c} \begin{array}{c} \theta_{2}=a\;\text{tan}\;2(\left(-k_{1}a_{3}\;\text{sin}\;\theta_{3}+k_{2}\left(a_{2}+a_{3}\;\text{cos}\;\theta_{3}\right)\right)/\left({a_{2}}^{2}+{a_{3}}^{2}+2a_{2}a_{3}\;\text{cos}\;\theta_{3}\right),\\ \left(k_{1}\left(a_{2}+a_{3}\;\text{cos}\;\theta_{3}\right)+k_{2}a_{3}\;\text{sin}\;\theta_{3}\right)/\left({a_{2}}^{2}+{a_{3}}^{2}+2a_{2}a_{3}\;\text{cos}\;\theta_{3}\right)) \end{array} \end{array}$$ (24)

Finally, the last joint angle *θ*_4_ can be obtained as

$$\begin{array}{c} \theta_{4}=\theta_{234}-\theta_{2}-\theta_{3} \end{array}$$ (25)

## C Proof of Theorem 1

In the point to point trajectory planing based on polynomial interpolation, the planning time is in inverse proportion to the maximum velocity of planning trajectory.

From common sense, since the distance between start knot and final knot is constant, if the planning time increases, then the average velocity and maximum velocity will decrease generally.

In theory, take a point to point trajectory planing based on third order polynomial interpolation as an example, and assume that there are two planning time (*t*_1_, *t*_2_ and *t*_1_ < *t*_2_) for the given trajectory, then the maximum velocity with planning time *t*_1_ is larger than that with planning time *t*_2_.

**Fig 16:**
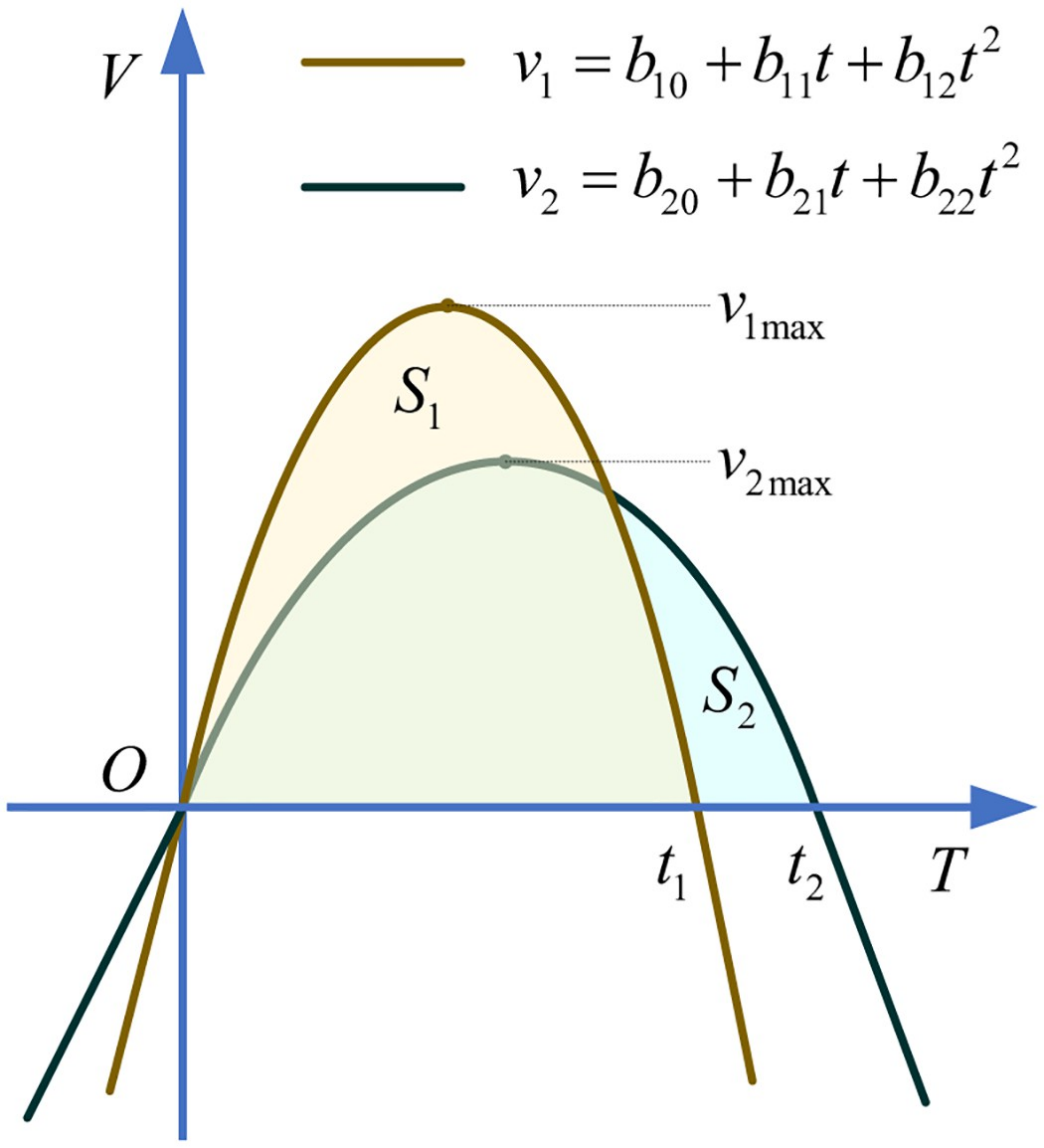
The velocity curves of trajectory planing based on third order polynomial interpolation with planning time *t*_1_ and *t*_2_.

To prove it, the detailed geometric description can be shown in Fig 16, where, *v*_1_ = *b*_10_ + *b*_11_*t* + *b*_12_*t*^2^ and *v*_2_ = *b*_20_ + *b*_21_*t* + *b*_22_*t*^2^ are the velocities of trajectory planing based on third order polynomial interpolation with planning time *t*_1_ and *t*_2_, respectively, *v*_1max_, *v*_2max_ are the maximum velocities of *v*_1_, *v*_2_, respectively, *S*_1_, *S*_2_ are the enclosure areas between the horizontal axis *T* and curves *v*_1_, *v*_2_, respectively, which both represent the constant distance between start knot and final knot.

As thus, the proposition can be set as follow.

**In the point to point trajectory planing based on third order polynomial interpolation, if** *t*_1_ < *t*_2_, **then** *v*_1 max_ > *v*_2 max_.

**Proof**: Assume that *t*_2_ = λ*t*_1_, λ > 1. Since *S*_1_ = *S*_2_, then it yields

$$\begin{array}{c} \begin{array}{cc} & S_{1}=S_{2}\\ \Rightarrow & \int_{0}^{t_{1}}v_{1}=\int_{0}^{t_{2}}v_{2}\\ \Rightarrow & \int_{0}^{t_{1}}b_{10}+b_{11}t+b_{12}t^{2}=\int_{0}^{t_{2}}b_{20}+b_{21}t+b_{22}t^{2}\\ \Rightarrow & b_{10}t+\frac{1}{2}b_{11}t^{2}+\frac{1}{3}b_{12}t^{3}{|}_{0}^{t_{1}}=b_{20}t+\frac{1}{2}b_{21}t^{2}+\frac{1}{3}b_{22}t^{3}{|}_{0}^{t_{2}}\\ \Rightarrow & b_{10}t_{1}+\frac{1}{2}b_{11}t_{1}^{2}+\frac{1}{3}b_{12}t_{1}^{3}=b_{20}t_{2}+\frac{1}{2}b_{21}t_{2}^{2}+\frac{1}{3}b_{22}t_{2}^{3}\\ \Rightarrow & b_{10}t_{1}+\frac{1}{2}b_{11}t_{1}^{2}+\frac{1}{3}b_{12}t_{1}^{3}=b_{20}\lambda t_{1}+\frac{1}{2}b_{21}\lambda^{2}t_{1}^{2}+\frac{1}{3}b_{22}\lambda^{3}t_{1}^{3}\\ \Rightarrow & b_{10}=b_{20}\lambda ,b_{11}=b_{21}\lambda^{2},b_{12}=b_{22}\lambda^{3} \end{array} \end{array}$$ (26)

From the 16, the maximum values of *v*_1_, *v*_2_ can be respectively written as

$$\begin{array}{c} \begin{array}{c} v_{1\;\text{max}}=\frac{4b_{12}b_{10}-b_{11}^{2}}{4b_{12}}\\ v_{2\;\text{max}}=\frac{4b_{22}b_{20}-b_{21}^{2}}{4b_{22}} \end{array} \end{array}$$ (27)

Combining (26) and (27), it yields

$$\begin{array}{c} \begin{array}{c} v_{1\;\text{max}}=\frac{4b_{12}b_{10}-b_{11}^{2}}{4b_{12}}\\ =\frac{4b_{22}\lambda^{3}b_{20}\lambda -b_{21}^{2}\lambda^{4}}{4b_{22}\lambda^{3}}\\ =\lambda \frac{4b_{22}b_{20}-b_{21}^{2}}{4b_{22}}\\ =\lambda v_{2\;\text{max}} \end{array} \end{array}$$ (28)

Since *t*_2_ = λ*t*_1_, λ > 1, then *v*_1 max_ > *v*_2 max_.

The trajectory planing based on other *n*-th order, including the seventh order polynomial interpolation, can be analyzed in the same way and the same conclusion can be obtained.

## D Proof of Theorem 2

Assume that the system state equation can be written as

$$\begin{array}{c} \begin{array}{c} {\dot{x}}_{1}=x_{2}\\ y=x_{2}=u(T) \end{array} \end{array}$$ (29)

where *x*_1_ donates the maximum velocity of trajectory planning. For the system, the desired output is *x*_1*d*_, which is the same as maximum velocity of actuator specifications, and *u*(*T*) is the control input with respect to planning time *T*. The control objective is to obtain an optimal planning time *T*_*opt*_ to make the maximum velocity of trajectory planning equal to the maximum velocity of actuator specifications.

Set the error between the maximum velocity of trajectory planning and the maximum velocity of actuator specifications as

$$\begin{array}{c} e=x_{1}-x_{1d} \end{array}$$ (30)

then it yields

$$\begin{array}{c} \dot{e}={\dot{x}}_{1}-{\dot{x}}_{1d} \end{array}$$ (31)

Let the nonnegative Lyapunov function as

$$\begin{array}{c} V=\frac{1}{2}e^{2} \end{array}$$ (32)

then combining Eqs (29) and (31) yields

$$\begin{array}{c} \begin{array}{c} \dot{V}=e\dot{e}\\ =e({\dot{x}}_{1}-{\dot{x}}_{1d})\\ =e(x_{2}-{\dot{x}}_{1d})\\ =e(u(T)-{\dot{x}}_{1d}) \end{array} \end{array}$$ (33)

Since ${\dot{x}}_{1d}=0$, then the control input can be written in P control form as

$$\begin{array}{c} u(T)={\dot{x}}_{1d}-k_{p}e=-k_{p}e \end{array}$$ (34)

where *k*_*p*_ is the coefficient of P control.

And then,

$$\begin{array}{c} \begin{array}{c} \dot{V}=e(u(T)-{\dot{x}}_{1d})\\ =e({\dot{x}}_{1d}-k_{p}e-{\dot{x}}_{1d})\\ =-k_{p}e^{2} \end{array} \end{array}$$ (35)

Therefore, the P control could guarantee the stability of time optimal controller and the converge of optimization. The maximum acceleration term in the conditional P control Eq (6) can be deduced in the same way. By adding the event-trigger, this is the reason why condition P control is employed.
